# Addition of inflammation-related biomarkers to the CAIDE model for risk prediction of all-cause dementia, Alzheimer’s disease and vascular dementia in a prospective study

**DOI:** 10.1186/s12979-024-00427-2

**Published:** 2024-04-03

**Authors:** Kira Trares, Manuel Wiesenfarth, Hannah Stocker, Laura Perna, Agnese Petrera, Stefanie M. Hauck, Konrad Beyreuther, Hermann Brenner, Ben Schöttker

**Affiliations:** 1 Division of Clinical Epidemiology and Aging Research, German Cancer Research Center, Im Neuenheimer Feld 581, Heidelberg, 69120 Germany; 2 Division of Biostatistics, German Cancer Research Center, Heidelberg, Germany; 3 Department of Genes and Environment, Max Planck Institute of Psychiatry, Kraepelinstraße 2-10, Munich, 80804 Germany; 4 Division of Mental Health of Older Adults, Department of Psychiatry and Psychotherapy, University Hospital, LMU Munich, Munich, 80336 Germany; 5 Metabolomics and Proteomics Core, Helmholtz Zentrum München, German Research Center for Environmental Health (GmbH), Neuherberg, Germany; 6 Network Aging Research, Heidelberg University, Bergheimer Straße 20, Heidelberg, 69115 Germany

**Keywords:** Inflammation, Risk prediction, Cohort study, Dementia, Alzheimer’s disease, Vascular dementia

## Abstract

**Background:**

It is of interest whether inflammatory biomarkers can improve dementia prediction models, such as the widely used Cardiovascular Risk Factors, Aging and Dementia (CAIDE) model.

**Methods:**

The Olink Target 96 Inflammation panel was assessed in a nested case-cohort design within a large, population-based German cohort study (*n* = 9940; age-range: 50–75 years). All study participants who developed dementia over 20 years of follow-up and had complete CAIDE variable data (*n* = 562, including 173 Alzheimer’s disease (AD) and 199 vascular dementia (VD) cases) as well as *n* = 1,356 controls were selected for measurements. 69 inflammation-related biomarkers were eligible for use. LASSO logistic regression and bootstrapping were utilized to select relevant biomarkers and determine areas under the curve (AUCs).

**Results:**

The CAIDE model 2 (including Apolipoprotein E (*APOE*) ε4 carrier status) predicted all-cause dementia, AD, and VD better than CAIDE model 1 (without *APOE* ε4) with AUCs of 0.725, 0.752 and 0.707, respectively. Although 20, 7, and 4 inflammation-related biomarkers were selected by LASSO regression to improve CAIDE model 2, the AUCs did not increase markedly. CAIDE models 1 and 2 generally performed better in mid-life (50–64 years) than in late-life (65–75 years) sub-samples of our cohort, but again, inflammation-related biomarkers did not improve their predictive abilities.

**Conclusions:**

Despite a lack of improvement in dementia risk prediction, the selected inflammation-related biomarkers were significantly associated with dementia outcomes and may serve as a starting point to further elucidate the pathogenesis of dementia.

**Supplementary Information:**

The online version contains supplementary material available at 10.1186/s12979-024-00427-2.

## Introduction

The number of dementia cases worldwide is continuously rising and is projected to double nearly every 20 years [1]. With the approval of *Aduhelm, Leqembi, and Donanemab* as the first effective treatments against Alzheimer’s disease (AD) by the U.S. Food and Drug Administration (FDA) there is hope for significant advancements in AD therapy. Although the drugs' efficacy, safety, and clinical application are still controversial [2–5], they can be considered a first step towards an effective dementia treatment. The above and future improved drugs will likely be most effective in early AD treatment. Thus, it is vital to perform dementia risk assessments and make diagnoses early [6, 7].

The scientific literature on dementia risk prediction increased rapidly since new risk factors and biomarkers were identified during the last years. However, sample sizes and follow-up durations varied extremely, and external validation is often lacking [6]. Also, the underlying study populations are highly different. Risk prediction models combining demographic, cognition, physical and health risk factors are often best suited and versatile [8, 9]. The Cardiovascular Risk Factors, Aging and Dementia (CAIDE) model, which is based on data from a Finnish population-based study, is such a risk model [10]. Including several risk factors of dementia, the authors could predict the risk of developing dementia with an area under the curve (AUC) of 0.769 (95% confidence interval (CI): 0.709 – 0.829). A second model containing additionally Apolipoprotein E (*APOE*) ε4 performed slightly better (AUC [95% CI]: 0.776 [0.717 – 0.836]). The CAIDE model was internally and externally validated in many cohorts, including high-income countries and various ethnicities [11–15]. However, the performance of the model was attenuated when applied to low-income countries as well as late-life cohorts [16, 17].

Dementia prediction models, including the CAIDE model, do not contain inflammatory biomarkers, although inflammation is a critical mechanism contributing to dementia pathogenesis [18]. Previously, we showed that most of the 92 inflammation-related biomarkers of the Olink Target 96 inflammation panel were significantly associated with all-cause dementia [19].

In this study, we fitted the CAIDE model to a large prospective cohort study and aimed to assess the potential of improving its ability to predict dementia risk by including inflammation-related biomarkers. Different models for all-cause dementia, AD, and vascular dementia (VD) as well as a mid-life and late-life population, were created.

## Methods

### Study population

This study was based on data from the ESTHER study. The ESTHER study (Epidemiologische Studie zu Chancen der Verhütung, Früherkennung und optimierten Therapie chronischer Erkrankungen in der älteren Bevölkerung [German]) is a prospective cohort study conducted in Saarland, Germany. Participants were recruited during a general health checkup at their general practitioners (GP) between 2000 and 2002 and were followed up 2, 5, 8, 11, 14, 17, and 20 years after baseline. The study comprises 9940 men and women between 50 and 75 years. Details have been described elsewhere [20]. Sociodemographic baseline characteristics were similarly distributed in the respective age categories as in a German National Health Survey conducted in a representative sample of the German population around the time of recruitment [20]. The study was approved by the ethics committees of the Medical Faculty of Heidelberg and the state medical board of Saarland, Germany.

### Dementia ascertainment and case-cohort design sample

**Fig. 1:**
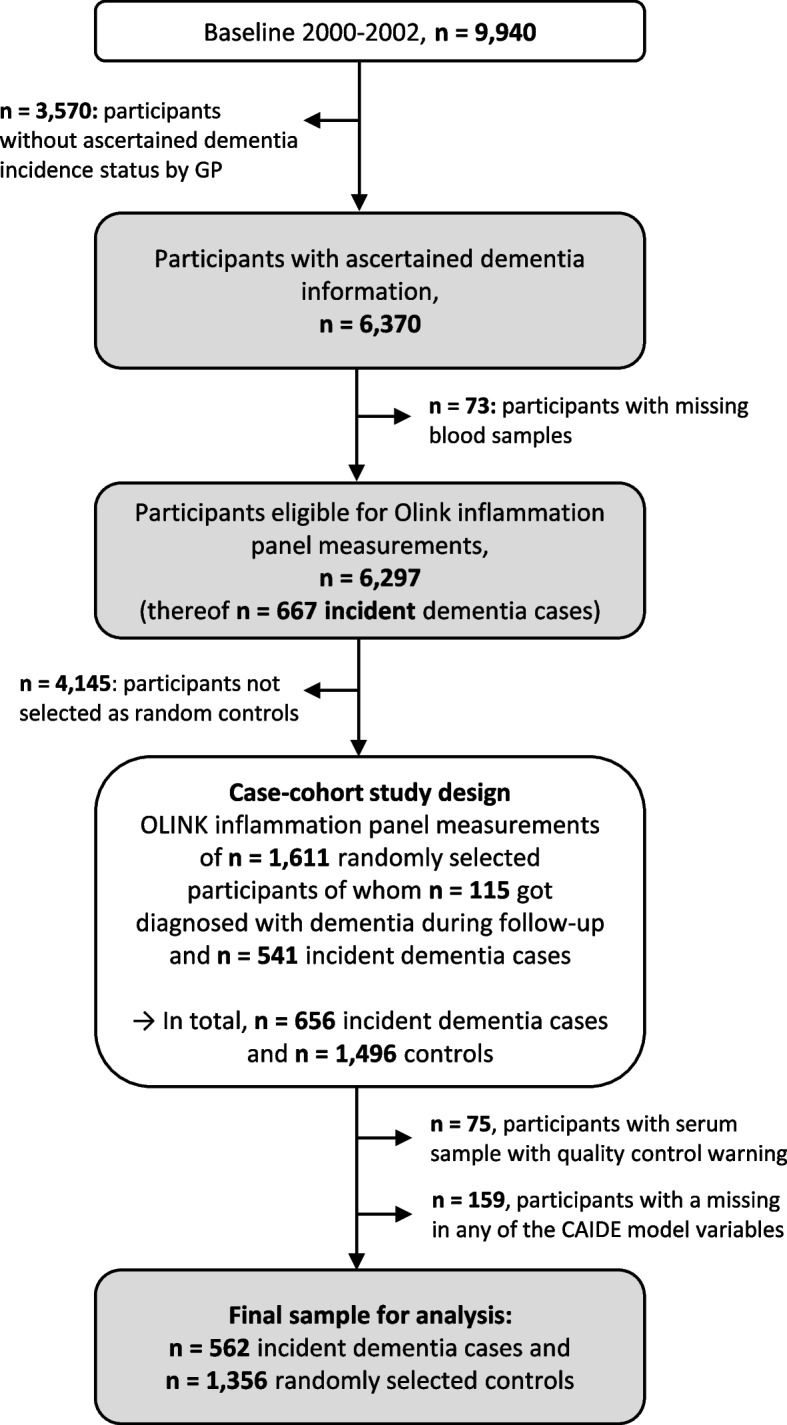
Flowchart of dementia ascertainment during the 14-, 17-, and 20-year follow-up of the ESTHER study and study participant selection. Abbreviations: GP General practitioner

Dementia information was collected during the 14-, 17-, and 20-year follow-up (median (interquartile range) follow-up time: 16.3 years (13.5–17.0 years)) via standardized questionnaires sent to the GPs of the ESTHER study’s participants. In this questionnaire, the GPs were asked whether dementia has been diagnosed among their patients and, if so, to provide all medical records from neurologists, psychiatrists, memory clinics, or other specialized providers. This query was also sent to the GPs of study participants who had already dropped out due to ill health or death. Overall, information on whether dementia was diagnosed during 20 years of follow-up or not could be ascertained for *n* = 6,466 study participants (65% of the original cohort). A flowchart of the study population is shown in Fig. 1.

After excluding subjects with missing blood samples (*n* = 73) from participants with ascertained dementia information, 6,297 participants were eligible to be drawn for the case-cohort sample and measurements of the Olink Target 96 inflammation panel. The randomly selected sample consisted of 1,611 study participants, of whom 115 were diagnosed with dementia during follow-up. Among the remaining 4,686 study participants not randomly selected, 541 were incident dementia cases and added to the data set as well, resulting in 656 dementia cases overall. However, due to quality control warnings during the biomarker measurements, 75 participants were additionally excluded. Participants with missing data for any of the aforementioned CAIDE model variables were further excluded (*n* = 159). For the last exclusion step, we compared the data of included and excluded participants with respect to age, sex, and education, and no indication of selection bias was detected (Supplemental Table 1). The final sample included a total of 562 dementia cases and 1,356 controls.

### Origin, assessment and modifications of the CAIDE model

The CAIDE model originates from the CAIDE study, a population-based cohort study from Finland assessing cardiovascular risk factors, aging, and dementia [21]. For the development of the CAIDE model, 1,409 participants aged between 39 and 64 years of the original CAIDE study were included [10]. Of those, 61 developed dementia during 20 years of follow-up. CAIDE model 1 consists of the variables age, education, sex, systolic blood pressure, body mass index (BMI), total cholesterol, and physical activity, while CAIDE model 2 additionally includes *APOE* ε4 status.

In the ESTHER study, the CAIDE model variables age, sex, education, body mass index (BMI), and physical activity of participants were assessed during the baseline assessment by standardized self-administered questionnaires. The systolic blood pressure of participants was measured at baseline by the GP. Total cholesterol levels were measured from serum samples by an enzymatic colorimetric test with the Synchron LX multicalibrator system (Beckman Coulter, Galway, Ireland). *APOE* genotypes were determined by TaqMan single-nucleotide polymorphism (SNP) genotyping assays (Applied Biosystems, California, USA). Endpoint allelic discrimination reads were used to analyze genotypes with the Bio-RAD CFX Connect System (Bio-Rad Laboratories, CA, USA). In the case of missing directly genotyped *APOE* data (*n* = 70), imputed quality-controlled data was used. For details, see Stocker et al. 2020 [22].

All variables used in the CAIDE model were available but it needed to be newly calibrated because the ESTHER cohort has a different age range, school education history and physical activity assessment than the CAIDE study. Fractional polynomials were utilized to determine the best fitting function of the continuous variables in the prediction of all-cause dementia, AD, and VD [23] (data not shown). Because the linear function was the best fitting for systolic blood pressure and BMI, they were kept as continuous variables. Although the best fitting function was x^(−2)^ for age and total cholesterol for all-cause dementia and VD, they were still modelled with the linear function because the difference in model fit was small. Education, physical activity, and *APOE* genotypes were dichotomized by summarizing categories with very similar odds ratios (ORs) for the association with all-cause dementia (data not shown).

### Measurement of inflammation-related biomarkers

Levels of inflammation-related proteins were measured in baseline serum samples using the Olink Target 96 inflammation panel (Olink Proteomics, Uppsala, Sweden). Details are described in Supplemental Text 1. In addition, a list of all biomarkers is depicted in Supplemental Table 2.

### Statistical analyses

The associations of the CAIDE model variables with the outcomes of all-cause dementia, AD, and VD were determined by a multivariate logistic regression model adjusted for age, education, sex, systolic blood pressure, BMI, total cholesterol, physical activity, and *APOE* ε4 status.

The predictive accuracy of the CAIDE model, including baseline variables and the inflammatory biomarkers measured from baseline serum samples, was assessed for dementia diagnoses collected over 20 years of follow-up, using least absolute shrinkage and selection operator (LASSO) logistic regression models. LASSO is a form of linear regression that uses shrinkage to exclude variables that are not useful for the prediction [24]. This makes the final equation simpler and easier to interpret. The CAIDE model variables were defined as not being penalized by the LASSO regression and thus forced into the model. In a sensitivity analysis, all variables were penalized. The parameter λ was determined by five-fold cross-validation. The AUCs and 95% CIs were estimated using 500 bootstrap samples for the CAIDE model and CAIDE model + inflammatory biomarkers for all-cause dementia, AD, and VD as the outcome, respectively. While the CAIDE model only included the CAIDE model variables, the CAIDE model + inflammatory biomarkers additionally included those of the 69 inflammation-related biomarkers selected by the LASSO regression. Moreover, we distinguished CAIDE models 1 and 2, with only the latter including *APOE* ε4 carrier status among the unpenalized CAIDE model variables. To determine if the differences between the CAIDE model and the CAIDE model + inflammatory biomarkers models were statistically significant, bootstrap intervals for the differences in AUCs were computed. This involves the calculation of the AUC difference between the two models for every bootstrap sample, sorting and assessing the true AUC difference. The probability of a variable to be selected by the LASSO regression was additionally determined using bootstrap inclusion frequencies [25, 26], providing insights about the number of selections for each variable throughout the bootstrapping procedure. High inclusion frequencies indicate a continuous impact on the model’s performance by the respective variables.

Besides calculations for the total sample, the models' discrimination performance was also evaluated in subgroups for mid-life (50–64 years) and late-life (65–75 years) for all three dementia outcomes and CAIDE model 1 and CAIDE model 2.

The Statistical Analysis System (SAS, version 9.4, Cary, North Carolina, USA) was used for multivariate logistic regression. Statistical tests were two-sided, using an alpha level of 0.05. LASSO regression was performed using the R package “*glmnet”* (R, version 3.6.3; glmnet package version 4.1–2) [27]. For AUC computation and bootstrapping, the R package *ModelGood* (R, version 3.6.3; ModelGood package version 1.0.9) was used [28].

## Results

Table 1 shows the CAIDE model variables of all included study participants separately for all-cause dementia (*n* = 562), AD (*n* = 173), and VD (*n* = 199) cases, as well as healthy controls (*n* = 1356). Most all-cause dementia cases were represented in the late-life sub-sample (63.2%). Furthermore, a larger proportion of subjects among controls had a higher school education than the basic education of 9 years (23.6%) than among the all-cause dementia cases (20.3%). Slightly more females than males were included in both cases (53.7%) and controls (54.7). Mean values for systolic blood pressure, BMI, and total cholesterol levels were comparable between all-cause dementia cases and controls. In addition, all-cause dementia cases included a higher proportion of physically inactive participants (26.0% compared to 17.6%) and a much higher proportion of *APOE* ε4 carriers than controls (39.5% compared to 24.3%). In a multivariate logistic regression model, only age, total cholesterol (inversely), physical activity (inversely) and *APOE* genotype were statistically significantly associated with all-cause dementia (Supplemental Table 3). In the model for AD (Supplemental Table 4), BMI was additionally significant and total cholesterol lost statistical significance in CAIDE model 1. In the model for VD (Supplemental Table 5), physical activity was not statistically significant. Age and *APOE* genotype were statistically significantly associated with all dementia outcomes.

**Table 1:** CAIDE model variables of included participants (*n*=1,918)

CAIDE model variables	Controls (*n* =1356)	Cases		
		All-cause dementia (*n* =562)	Alzheimer’s disease (*n*=173)	Vascular dementia (*n*=199)
Age (years), mean (SD)	61.7 (6.5)	66.3 (5.2)	66.3 (5.1)	66.5 (5.1)
Mid-life (50-64 years), *n* (%)	867 (63.9)	207 (36.8)	65 (37.6)	68 (34.2)
Late-life (65-75 years), *n* (%)	489 (36.1)	355 (63.2)	108 (62.4)	131 (65.8)
Education (years), mean (SD)				
≤ 9	1036 (76.4)	448 (79.7)	141 (81.5)	160 (80.4)
> 9	320 (23.6)	114 (20.3)	32 (18.5)	39 (19.6)
Sex, *n* (%)				
Female	742 (54.7)	302 (53.7)	97 (56.1)	105 (52.8)
Male	614 (45.3)	260 (46.3)	76 (43.9)	94 (47.2)
SBP (mmHg), mean (SD)	138.9 (19.1)	142.2 (19.4)	142.2 (19.3)	141.9 (19.8)
BMI (kg/m^2^), mean (SD)	27.8 (4.4)	27.5 (3.9)	27.2 (3.7)	27.6 (3.9)
Total cholesterol (mmol/L), mean (SD)	5.9 (1.23)	5.7 (1.3)	5.7 (1.3)	5.7 (1.4)
Physical activity ^a^, *n* (%)				
Inactive	239 (17.6)	146 (26.0)	53 (30.6)	49 (24.6)
Active	1117 (82.4)	416 (74.0)	120 (69.4)	150 (75.4)
*APOE* genotype, *n* (%)				
ε4 non-carrier	1027 (75.7)	340 (60.5)	89 (51.5)	128 (64.3)
ε4 carrier	329 (24.3)	222 (39.5)	84 (48.5)	71 (35.68)

*Abbreviations*: *APOE* Apolipoprotein E, *SBP* Systolic blood pressure, *BMI* Body mass index

^a^“Inactive” was defined as <1 hour of vigorous or <1 hour of light physical activity per week. All other amounts of physical activity were grouped into the category “Active”

**Fig. 2:**
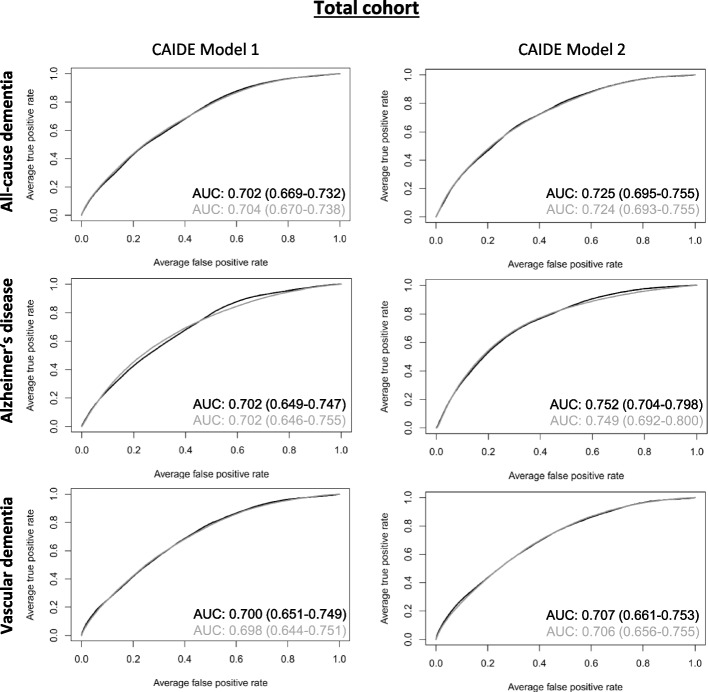
ROC curves of created all-cause dementia, Alzheimer’s disease, and vascular dementia risk prediction models for the total cohort. ROC curves for CAIDE model 1 (including age, education, sex, systolic blood pressure, BMI, total cholesterol, and physical activity, and CAIDE model 2 (additionally including *APOE* ε4 carrier status) are depicted in black while curves of the CAIDE models plus inflammatory biomarkers chosen by LASSO regression (cf. Table 3) are depicted in grey. AUC and 95% bootstrap confidence intervals are provided with the respective graphs. The AUCs were obtained in a nested case-cohort study with *n* = 1,356 healthy controls and *n* = 562, *n* = 173, and *n* = 199 cases for all-cause dementia, Alzheimer’s disease, and vascular dementia, respectively. Abbreviations: BMI Body mass index, APOE Apolipoprotein, LASSO Least absolute shrinkage and selection operator

Table 2 shows the discriminative performances of various prediction models for all-cause dementia, AD, and VD. All CAIDE models had a high discriminative performance in the total cohort with an AUC ≥ 0.7 (Fig. 2). However, inflammatory biomarkers selected by the LASSO logistic regression did not improve the models’ discriminative performance. The inflammation-related biomarkers selected by LASSO regression are shown in Table 3. In total, 20, 7, and 4 inflammatory biomarkers were added to the CAIDE model 2 for all-cause dementia, AD, and VD, respectively. The selected biomarkers differed between the outcomes but were similar for CAIDE model 1 and 2 for each outcome. The β-coefficients of all variables needed to calculate risk scores for the CAIDE + inflammatory biomarkers models and bootstrap inclusion frequencies for all-cause dementia, AD and VD can be found in Supplemental Tables 6, 7, 8 respectively. Bootstrap inclusion frequencies showed a relatively clear cutoff for variables selected by LASSO compared to non-selected ones (data not shown).

**Table 2:** Discrimination performance of models

		**n**_**total**_	**n**_**cases**_	**CAIDE Model 1**^**a**^		**CAIDE Model 2**^**b**^	
				**AUC (95% CI)**	**∆ AUC (95% CI)**^**c**^	**AUC (95% CI)**	**∆ AUC (95% CI)**^**c**^
**Total cohort**	**All-cause dementia**						
	CAIDE Model	1918	562	0.702 (0.669-0.732)	-	0.725 (0.695-0.755)	-
	CAIDE Model + inflam. biomarkers^d^			0.704 (0.670-0.738)	0.001 (-0.030-0.021)	0.724 (0.693-0.755)	-0.001 (-0.027-0.019)
	**Alzheimer’s disease**						
	CAIDE Model	1529	173	0.702 (0.649-0.747)	-	0.752 (0.704-0.798)	-
	CAIDE Model + inflam. biomarkers^d^			0.702 (0.646-0.755)	0.000 (-0.051-0.038)	0.749 (0.692-0.800)	-0.002 (-0.049-0.029)
	**Vascular dementia**						
	CAIDE Model	1555	199	0.700 (0.651-0.749)	-	0.707 (0.661-0.753)	-
	CAIDE Model + inflam. biomarkers^d^			0.698 (0.644-0.751)	-0.002 (-0.050-0.0327)	0.706 (0.656-0.755)	-0.001 (-0.047-0.039)
**Mid-Life (50-64 years)**	**All-cause dementia**						
	CAIDE Model	1074	207	0.697 (0.652-0.743)	-	0.721 (0.673-0.769)	-
	CAIDE Model + inflam. biomarkers^e^			0.701 (0.646-0.748)	0.004 (-0.044-0.040)	0.718 (0.665-0.768)	-0.003 (-0.051-0.035)
	**Alzheimer’s disease**						
	CAIDE Model	932	65	0.700 (0.619-0.783)	-	0.751 (0.678-0.830)	-
	CAIDE Model + inflam. biomarkers^e^			0.690 (0.602-0.772)	-0.010 (-0.091-0.050)	0.737 (0.656-0.820)	-0.014 (-0.087-0.036)
	**Vascular dementia**						
	CAIDE Model	935	68	0.665 (0.587-0.740)	-	0.672 (0.589-0.750)	-
	CAIDE Model + inflam. biomarkers^e^			0.691 (0.610-0.762)	0.026 (-0.062-0.096)	0.693 (0.608-0.767)	0.021 (-0.083-0.089)
**Late-life (65-75 years)**	**All-cause dementia**						
	CAIDE Model	844	355	0.582 (0.535-0.633)	-	0.624 (0.570-0.676)	-
	CAIDE Model + inflam. biomarkers^f^			0.576 (0.528-0.626)	-0.006 (-0.066-0.039)	0.609 (0.556-0.668)	-0.014 (-0.069-0.024)
	**Alzheimer’s disease**						
	CAIDE Model	597	108	0.575 (0.473-0.655)	-	0.651 (0.566-0.724)	-
	CAIDE Model + inflam. biomarkers^f^			0.595 (0.492-0.676)	0.021 (-0.087-0.109)	0.650 (0.561-0.725)	-0.001 (-0.084-0.061)
	**Vascular dementia**						
	CAIDE Model	620	131	0.558 (0.465-0.637)	-	0.582 (0.488-0.665)	-
	CAIDE Model + inflam. biomarkers^f^			0.547 (0.460-0.629)	-0.010 (-0.100-0.074)	0.556 (0.475-0.641)	-0.026 (-0.126-0.043)

*Abbreviations*: inflam Inflammatory, AUC Area under the curve, CI Confidence interval

^a^The CAIDE model 1 includes age, education, sex, systolic blood pressure, body-mass index, total cholesterol and physical activity

^b^The CAIDE model 2 includes the variables of CAIDE model 1 and *APOE* ε4 status

^c^The 95% CI is the bootstrap interval for the differences in AUCs.

^d^The inflammatory biomarkers selected by the LASSO regression are shown in Table 3

^e^The inflammatory biomarkers selected by the LASSO regression for all-cause dementia, Alzheimer’s disease and vascular dementia are shown in Suppl. Tables 9, 10 and 11, respectively

^f^The inflammatory biomarkers selected by the LASSO regression for all-cause dementia, Alzheimer’s disease and vascular dementia are shown in Suppl. Tables 12, 13 and 14, respectively

**Table 3:** Inflammatory biomarkers selected by LASSO regression in the total cohort (*n*=1,918)

Inflammatory biomarkers	Improvement of the CAIDE models’ predictive ability for dementia outcomes		
	All-cause dementia	Alzheimer’s disease	Vascular dementia
4E BP1	Model 2	-	-
Beta_NGF	Model 1+2	-	-
CCL23	Model 1+2	-	-
CCL3	Model 2	-	-
CD244	Model 1+2	-	Model 1+2
CST5	-	Model 1+2	-
CXCL1	Model 1+2	-	-
CXCL5	Model 1+2	-	-
EN-RAGE	Model 1+2	Model 1+2	Model 1+2
FGF21	Model 1	-	-
IL18	Model 1+2	-	Model 1+2
IL7	Model 2	Model 2	-
LAP TGF beta1	Model 1+2	Model 1+2	Model 1+2
LIFR	Model 1+2	-	-
MCP3	Model 2	Model 2	-
MMP1	-	Model 2	-
OPG	Model 1+2	-	-
OSM	Model 1+2	-	-
SCF	Model 2	-	-
SIRT2	Model 2	-	-
SLAMF1	Model 1+2	-	-
TNFB	Model 1+2	-	-
TRAIL	-	Model 2	-
VEGFA	Model 1+2	-	-

Model 1 and Model 2 refer to CAIDE Model 1 and CAIDE Model 2, respectively

*Abbreviations*: For inflammatory biomarker abbreviations, see Supplemental Table 2

The prediction of CAIDE model 2 improved more for AD and all-cause dementia than VD compared to CAIDE model 1. Overall, the highest discriminative performance of all models was achieved for AD for CAIDE model 2 without inflammatory biomarkers (AUC [95% CI]: 0.752 [0.704–0.798]).

In a further step, we split the cohort into a mid-life (50–64 years) and late-life (65–75 years) sub-sample. A clear difference in dementia prediction between the age groups became apparent (Table 2**,** Supplemental Figs. 1  and 2). While the AUCs for the various models for all-cause dementia, AD, and VD varied between 0.665 and 0.751 in the mid-life sample, AUCs in the late-life sample were consistently lower and ranged between 0.547 and 0.651. Inflammatory biomarkers selected by the LASSO regression did not lead to improvements in the models' AUCs, neither in the mid-life nor the late-life subsample. The inflammatory biomarkers selected by the LASSO regression and the β-coefficients for their associations with all-cause dementia, AD and VD, as well as the other CAIDE variables needed to calculate the risk prediction models and bootstrap inclusion frequencies, are shown in Supplemental Tables 9, 10, 11 for the mid-life and Supplemental Tables 12, 13, 14 for the late-life sample, respectively. Comparable to the total cohort, the highest AUCs were achieved for AD when the inflammatory biomarkers were not included in CAIDE model 2 (AUC [95% CI]: 0.751 [0.678–0.830] and 0.651 [0.566–0.724] for the mid-life and late-life sample, respectively).

In a sensitivity analysis, we penalized not only the OLINK inflammation biomarkers but also the variables of the CAIDE model 1 in the LASSO regression. This analysis was exemplarily conducted for CAIDE model 1 and the outcome of all-cause dementia. Interestingly, all CAIDE model variables except sex and education were selected, and the same list of inflammatory biomarkers with only one addition was chosen (CXCL5). In addition, the AUC of this sensitivity analysis (0.703 [0.674–0.734]) was almost identical to the one from the main analysis (0.702 [0.669–0.732]).

## Discussion

In this prospective cohort study, we aimed to explore the potential for improving the predictive ability of the CAIDE model by including the serum levels of inflammation-related proteins. Although several biomarkers were selected by LASSO regression to the CAIDE model for the prediction of all-cause dementia, AD, and VD, AUCs did not change. Nevertheless, these are still important findings in this research field.

### Previous studies

In previous studies, the CAIDE score showed good external validity in five cohorts without any adjustments to the model [11, 12, 14, 15, 29]. All of them reported a similar discriminative performance of the score. Moreover, a recent Cochrane review performed a meta-analysis on three studies externally validating the CAIDE model [30]. Overall the meta-analysis revealed a good predictive ability of the CAIDE model (AUC [95% CI]: 0.71 [0.66–0.76]). However, the authors expressed concerns about the certainty of the underlying data. Besides, the CAIDE risk score was evaluated as a tool for dementia risk prediction in different ethnicities and showed good predictive ability in subgroups for Asians and dark-skinned people [11]. However, the prognosis was poor in cohorts of Hispanic/Latino Americans and Japanese American men [13, 31]. Furthermore, Stephan and colleagues recently showed that the CAIDE score has poor predictive ability in low- and middle-income countries (0.52 ≤ c ≤ 0.63) [17]. Furthermore, a poor performance of the CAIDE model was observed in late-life samples in previous studies by Anstey and Kivimäki et al. [16, 29] and Fayosse et al. [12]. The latter showed that the CAIDE model only significantly predicted dementia at a mean age of 55 but not at 60 or 65 years, when examining participants separately. Thus, despite its unquestionable merits, improvements of the CAIDE score are needed.

To our knowledge, four modifications of the CAIDE score are available: Tolea and colleagues designed a modified version of the CAIDE score (mCAIDE) to simplify the application of the model in a community-based setting [32]. Therefore, laboratory measurements of cholesterol levels were replaced by self-reported information about high cholesterol levels (yes or no). In addition, physical activity assessment was replaced by the mini Physical Performance Testing (mPPT). The mCAIDE score was first applied to a cohort of 230 community-dwelling older adults in which it slightly improved the discrimination between cognitively impaired and unimpaired individuals (AUC mCAIDE: 0.78 [0.71–0.85], AUC CAIDE: 0.71 [0.61–0.80]). Afterwards, the score was additionally validated in an independent clinical cohort of 219 participants and demonstrated to discriminate well between different stages of dementia.

Exalto and colleagues aimed to improve the predictive performance of the CAIDE score by including diabetes mellitus, depressed mood, head trauma, central obesity, lung function, and smoking as additional mid-life risk factors [11]. However, the added variables did not improve its predictive abilities.

Harrison and colleagues tested if adding a composite score of two biomarkers of inflammation (interleukin-6 and C-reactive protein) and one of oxidative stress (homocysteine) to the CAIDE score would improve the ability to predict cognitive decline for study participants of two cohorts aged 85 years or older [33]. Adding the biomarkers to the CAIDE score increased the hazard ratio (HR) for comparison of a high- and low-risk group from 1.14 (95% CI: 0.64–2.03, *p* = 0.65) to 1.96 (1.27–3.42, *p* = 0.02) in the first cohort and from 1.64 (1.04–2.58, *p* = 0.03) to 1.89 (1.18–3.02, *p* = 0.08) in the second cohort.

Finally, Geethadevi and colleagues applied the CAIDE model and two other dementia risk prediction models to an Australian cohort study, compared their predictive ability, and created a hybrid model including several variables of all three models chosen by a machine learning algorithm [34]. The CAIDE model showed the lowest predictive ability for dementia of all models in this cohort of 3360 participants (AUC [95% CI]: 0.54 [0.49–0.58]). Nonetheless, the created hybrid model included all variables of the CAIDE model as well as history of head injury, depression, diabetes mellitus, smoking status, alcohol consumption, social activity, cognitive activity, fish intake, history of coronary artery disease (CAD), and *APOE* ε4. With this set of variables, the authors achieved an AUC of 0.80 (95% CI: 0.78–0.83). However, the hybrid model still lacks external validation.

### Interpretation of findings

Compared to the original CAIDE model, the predictive ability in our study was lower but still good (AUCs of 0.769 and 0.776 for CAIDE model 1 and 2 in the original study compared to 0.702 and 0.725, respectively, for all-cause dementia in our study). In agreement with previous studies, we also observed a better predictive ability of the CAIDE model in mid-life than in late-life [12, 16, 29]. However, since it is more important to have suitable dementia risk assessment tools in mid-life than in late-life this is not critical. Targeting dementia risk factors in mid-life has a greater potential to prevent or delay the onset of the disease.

Although inflammation is considered to have a crucial role in dementia pathogenesis [35, 36], the discriminative ability of the CAIDE model did not increase when the inflammation-related biomarkers were added – neither in the total sample nor in the mid-life nor late-life sub-sample. This suggests that the variables included in the CAIDE model are already strong dementia predictors capturing the predictive ability of inflammatory biomarkers because there is some conceptual overlap (e.g., between age and inflammation or between low physical activity and inflammation). Apart from this, due to the long follow-up duration in our study and the single measurement at baseline, it is possible that the biomarker measurements only reflect a beginning inflammatory response of the immune system to early dementia onset and are not predictive for clinical dementia diagnoses in the long run.

Despite the lack of an added predictive value by the biomarkers, these results are still important for this research field. First, they underscore the robustness of the CAIDE model, which already encompasses key risk factors for dementia. CAIDE model 2, which comprises the *APOE* ε4 carrier status, reached the highest AUC without including the inflammation-related biomarkers. This is essential information for researchers aiming to improve the predictive abilities of the CAIDE and other dementia risk prediction models since it might be more promising to spend the time and resources on testing biomarkers addressing other aspects of dementia etiology.

Moreover, the inflammatory biomarkers chosen by LASSO regression might shed more light on the biological mechanisms underlying dementia pathogenesis. Notably, EN-RAGE and latency-associated peptide transforming growth factor beta-1 (LAP TGF-beta 1) were among the biomarkers chosen by LASSO regression for all-cause dementia, AD, and VD. EN-RAGE also showed the highest and most consistent bootstrap inclusion frequencies of > 73% for all outcomes (total cohort). The biomarker vascular endothelial growth factor-A (VEGF-A) was additionally chosen for all-cause dementia. In a previous analysis with our case-cohort sample from the ESTHER study, we showed that these biomarkers were independently associated with at least one of the outcomes and discussed the potential mechanisms involving different aspects of dementia pathogenesis, namely neurodegeneration (EN-RAGE), amyloid beta (Aß) deposition (LAP TGF-beta 1), and blood brain barrier permeability (VEGF-A) [37].

### Strengths and limitations

This study is characterized by the prospective cohort design, a long follow-up period of 20 years, its large sample size and its representativeness of the German healthcare setting. In addition, appropriate measures were taken to prevent overfitting of the developed models by applying LASSO logistic regression and bootstrapping [24, 28].

In the ESTHER study, dementia diagnoses are collected in a community-based setting. Although, diagnoses were collected from medical records, a thourough assessment of subtypes is often lacking the community setting. This might also explain the comparatively low proportion of AD cases. However, the most important outcome for dementia risk assessment in the community setting is all-cause dementia. Moreover, due to a different age structure, education system, and physical activity assessment in the ESTHER study compared to the CAIDE study, the CAIDE model needed to be refitted. This hampers a direct comparison to the results of the CAIDE model. Due to cost reasons, biomarker measurements were conducted in a case-cohort study design rather than a cohort design using the entire study population. In addition, biomarker measurements could only be performed once in baseline blood samples rather than in follow-up samples. This limitation may have resulted in an underestimation of the AUC because the inflammation status could change during follow-up. Finally, the results of this study originate from a study population that comprises mainly of participants of European descent aged 50 to 75 years. Hence, the results might not be generalized to other populations.

## Conclusion

This large, prospective cohort study showed that adding inflammation-related, blood-based biomarkers to the CAIDE model does not improve the model’s discriminative ability for all-cause dementia, AD, or VD. Nevertheless, as previously shown, the biomarkers selected by LASSO regression were significantly associated with the assessed outcomes and could thus serve as a starting point to further elucidate the pathogenesis of dementia. Other factors, less conceptionally related to the variables already included in the CAIDE model, should be included in future studies to improve its predictive value.

### Supplementary Information

**Supplementary Material 1. **

## Data Availability

The data that support the findings of this study are not openly available due to reasons of sensitivity and are available from the corresponding author upon reasonable request. Data are located in controlled access data storage at the German Cancer Research Center.

## Abbreviations

AD: Alzheimer’s disease
APOE: Apolipoprotein E
Aß: Amyloid beta
AUC: Area under the curve
BMI: Body mass index
CAD: Coronary artery disease
CAIDE: Cardiovascular Risk Factors, Aging and Dementia
CI: Confidence interval
EN-RAGE: Protein S100-A12
ESTHER: Epidemiologische Studie zu Chancen der Verhütung, Früherkennung und optimierten Therapie chronischer Erkrankungen in der älteren Bevölkerung [German]
FDA: Food and Drug Administration
GP: General practitioners
HR: Hazard ratio
LAP TGF-beta-1: Latency-associated peptide transforming growth factor beta-1
LASSO: Least absolute shrinkage and selection operator
mCAIDE: Modified version of the CAIDE score
mPPT: Mini Physical Performance Testing
OR: Odds ratio
SNP: Single-nucleotide polymorphism
VD: Vascular dementia
VEGF-A: Vascular endothelial growth factor-A

## References

[1] Prince M, Wimo A, Guerchet M, Ali G-C, Wu Y-T, Prina M. World Alzheimer Report 2015 - The Global Impact of Dementia an Analysis of Prevalence, Incidence, Cost, and Trends. In: Alzheimers Dis Int. 2015. https://www.alzint.org/u/WorldAlzheimerReport2015.pdf. Accessed 07 Aug 2023.

[2] Mahase E (2021). Aducanumab: European agency rejects Alzheimer’s drug over efficacy and safety concerns. BMJ.

[3] Alexander GC, Emerson S, Kesselheim AS (2021). Evaluation of Aducanumab for Alzheimer Disease: Scientific Evidence and Regulatory Review Involving Efficacy, Safety, and Futility. JAMA.

[4] Perneczky R, Jessen F, Grimmer T, Levin J, Flöel A, Peters O (2023). Anti-amyloid antibody therapies in Alzheimer’s disease. Brain.

[5] Cummings J (2023). Anti-Amyloid Monoclonal Antibodies are Transformative Treatments that Redefine Alzheimer's Disease Therapeutics. Drugs.

[6] Hou X-H, Feng L, Zhang C, Cao X-P, Tan L, Yu J-T (2019). Models for predicting risk of dementia: a systematic review. J Neurol Neurosurg Psychiatry.

[7] Goerdten J, Čukić I, Danso SO, Carrière I, Muniz-Terrera G (2019). Statistical methods for dementia risk prediction and recommendations for future work: A systematic review. Alzheimer's & Dementia: Translational Research & Clinical Interventions.

[8] Tang EYH, Harrison SL, Errington L, Gordon MF, Visser PJ, Novak G (2015). Current Developments in Dementia Risk Prediction Modelling: An Updated Systematic Review. PLoS ONE.

[9] Stephan BCM, Kurth T, Matthews FE, Brayne C, Dufouil C (2010). Dementia risk prediction in the population: are screening models accurate?. Nat Rev Neurol.

[10] Kivipelto M, Ngandu T, Laatikainen T, Winblad B, Soininen H, Tuomilehto J (2006). Risk score for the prediction of dementia risk in 20 years among middle aged people: a longitudinal, population-based study. The Lancet Neurology.

[11] Exalto LG, Quesenberry CP, Barnes D, Kivipelto M, Biessels GJ, Whitmer RA (2014). Midlife risk score for the prediction of dementia four decades later. Alzheimers Dement.

[12] Fayosse A, Nguyen D-P, Dugravot A, Dumurgier J, Tabak AG, Kivimäki M (2020). Risk prediction models for dementia: role of age and cardiometabolic risk factors. BMC Med.

[13] Torres S, Alexander A, O'Bryant S, Medina LD (2020). Cognition and the Predictive Utility of Three Risk Scores in an Ethnically Diverse Sample. J Alzheimers Dis.

[14] Licher S, Yilmaz P, Leening MJG, Wolters FJ, Vernooij MW, Stephan BCM (2018). External validation of four dementia prediction models for use in the general community-dwelling population: a comparative analysis from the Rotterdam Study. Eur J Epidemiol.

[15] Virta JJ, Heikkilä K, Perola M, Koskenvuo M, Räihä I, Rinne JO (2013). Midlife cardiovascular risk factors and late cognitive impairment. Eur J Epidemiol.

[16] Anstey KJ, Cherbuin N, Herath PM, Qiu C, Kuller LH, Lopez OL (2014). A Self-Report Risk Index to Predict Occurrence of Dementia in Three Independent Cohorts of Older Adults: The ANU-ADRI. PLoS ONE.

[17] Stephan BCM, Pakpahan E, Siervo M, Licher S, Muniz-Terrera G, Mohan D (2020). Prediction of dementia risk in low-income and middle-income countries (the 10/66 Study): an independent external validation of existing models. Lancet Glob Health.

[18] Walker KA, Ficek BN, Westbrook R (2019). Understanding the Role of Systemic Inflammation in Alzheimer's Disease. ACS Chem Neurosci.

[19] Trares K, Bhardwaj M, Perna L, Stocker H, Petrera A, Hauck SM (2022). Association of the inflammation-related proteome with dementia development at older age: results from a large, prospective, population-based cohort study. Alzheimer's Research & Therapy.

[20] Stocker H, Beyer L, Trares K, Perna L, Rujescu D, Holleczek B (2023). Association of Kidney Function With Development of Alzheimer Disease and Other Dementias and Dementia-Related Blood Biomarkers. JAMA Netw Open.

[21] Kivipelto M, Helkala E-L, Hänninen T, Laakso MP, Hallikainen M, Alhainen K (2001). Midlife vascular risk factors and late-life mild cognitive impairment. A population-based study.

[22] Stocker H, Perna L, Weigl K, Möllers T, Schöttker B, Thomsen H (2021). Prediction of clinical diagnosis of Alzheimer’s disease, vascular, mixed, and all-cause dementia by a polygenic risk score and APOE status in a community-based cohort prospectively followed over 17 years. Molecular Psychiatry..

[23] Royston P, Sauerbrei W (2005). Building multivariable regression models with continuous covariates in clinical epidemiology–with an emphasis on fractional polynomials. Methods Inf Med.

[24] Tibshirani R (1996). Regression Shrinkage and Selection via the Lasso. J Roy Stat Soc: Ser B (Methodol).

[25] Heinze G, Wallisch C, Dunkler D (2018). Variable selection – A review and recommendations for the practicing statistician. Biom J.

[26] Sauerbrei W, Perperoglou A, Schmid M, Abrahamowicz M, Becher H, Binder H (2020). State of the art in selection of variables and functional forms in multivariable analysis—outstanding issues. Diagnostic and Prognostic Research.

[27] Friedman JH, Hastie T, Tibshirani R (2010). Regularization Paths for Generalized Linear Models via Coordinate Descent. J Stat Softw.

[28] Gerds TA. ModelGood: Validation of risk prediction models. R package version 1.0.9. ed2015. https://cran.r-project.org/web/packages/ModelGood/index.html.

[29] Kivimäki M, Livingston G, Singh-Manoux A, Mars N, Lindbohm JV, Pentti J (2023). Estimating Dementia Risk Using Multifactorial Prediction Models. JAMA Netw Open.

[30] Mohanannair Geethadevi G, Quinn TJ, George J, Anstey KJ, Bell JS, Sarwar MR, Cross AJ. Multi‐domain prognostic models used in middle‐aged adults without known cognitive impairment for predicting subsequent dementia. Cochrane Database Syst Rev. 2023;6:CD014885. 10.1002/14651858.CD014885.pub2 PMC10239281 37265424

[31] Chosy EJ, Edland SD, Gross N, Meyer MJ, Liu CY, Launer LJ (2019). The CAIDE Dementia Risk Score and the Honolulu-Asia Aging Study. Dement Geriatr Cogn Disord.

[32] Tolea MI, Heo J, Chrisphonte S, Galvin JE (2021). A Modified CAIDE Risk Score as a Screening Tool for Cognitive Impairment in Older Adults. J Alzheimers Dis.

[33] Harrison SL, de Craen AJM, Kerse N, Teh R, Granic A, Davies K (2017). Predicting Risk of Cognitive Decline in Very Old Adults Using Three Models: The Framingham Stroke Risk Profile; the Cardiovascular Risk Factors, Aging, and Dementia Model; and Oxi-Inflammatory Biomarkers. J Am Geriatr Soc.

[34] Geethadevi GM, Peel R, Bell JS, Cross AJ, Hancock S, Ilomaki J, et al. Validity of three risk prediction models for dementia or cognitive impairment in Australia. Age Ageing. 2022;51(12):afac307. 10.1093/ageing/afac307 PMC9804251 36585910

[35] Kinney JW, Bemiller SM, Murtishaw AS, Leisgang AM, Salazar AM, Lamb BT (2018). Inflammation as a central mechanism in Alzheimer's disease. Alzheimer's & dementia (New York, N Y).

[36] Raz L, Knoefel J, Bhaskar K (2016). The neuropathology and cerebrovascular mechanisms of dementia. J Cereb Blood Flow Metab.

[37] Trares K, Bhardwaj M, Perna L, Stocker H, Petrera A, Hauck SM (2022). Association of the inflammation-related proteome with dementia development at older age: results from a large, prospective, population-based cohort study. Alzheimer's Res Ther..

[38] Ranstam J, Cook JA (2018). LASSO regression. British J Surg.

