# Supplementary material for: Addition of inflammation-related biomarkers to the CAIDE model for risk prediction of all-cause dementia, Alzheimer’s disease and vascular dementia in a prospective study
Source: Immun Ageing. 2024 Apr 3;21:23. doi: 10.1186/s12979-024-00427-2 (PMC10988812; doi:10.1186/s12979-024-00427-2)
Supplement: Supplementary file 1 — Supplementary Material 1. [file 12979_2024_427_MOESM1_ESM.docx]

**Supplemental Material to Article**

“Improved risk prediction of all-cause dementia, Alzheimer’s disease, and vascular dementia by inflammation-related biomarkers”

Table of Contents

[Supplemental Figure 1. ROC curves of created all-cause dementia, Alzheimer’s disease and vascular dementia risk prediction models for the mid-life cohort. 2](#_Toc142463112)

[Supplemental Figure 2. ROC curves of created all-cause dementia, Alzheimer’s disease and vascular dementia risk prediction models for the late-life cohort. 4](#_Toc142463115)

[Supplemental Text 1. Quantative biomarker analysis 5](#_Toc142463117)

[Supplemental Table 1. Comparison of age, education, and sex of included and excluded study participants of the ESTHER study 7](#_Toc142463118)

[Supplemental Table 2. Biomarkers measured with Olink Proseek® Multiplex Inflammation I96x96 kits. 8](#_Toc142463119)

[Supplemental Table 3. Associations of CAIDE model variables with all-cause dementia. 10](#_Toc142463120)

[Supplemental Table 4. Associations of CAIDE model variables with Alzheimer’s disease. 11](#_Toc142463121)

[Supplemental Table 5. Associations of CAIDE model variables with vascular dementia. 12](#_Toc142463122)

[Supplemental Table 6. β-coefficients and bootstrap inclusion frequencies of variables included in prediction models for all-cause dementia in the total cohort 13](#_Toc142463123)

[Supplemental Table 7. β-coefficients and bootstrap inclusion frequencies of variables included in prediction models for Alzheimer’s disease in the total cohort 15](#_Toc142463124)

[Supplemental Table 8. β-coefficients and bootstrap inclusion frequencies of variables included in prediction models for vascular dementia in the total cohort 16](#_Toc142463125)

[Supplemental Table 9. β-coefficients and bootstrap inclusion frequencies of variables included in prediction models for all-cause dementia in the mid-life cohort 17](#_Toc142463126)

[Supplemental Table 10. β-coefficients and bootstrap inclusion frequencies of variables included in prediction models for Alzheimer’s disease in the mid-life cohort 18](#_Toc142463127)

[Supplemental Table 11. β-coefficients and bootstrap inclusion frequencies of variables included in prediction models for vascular dementia in the mid-life cohort 19](#_Toc142463128)

[Supplemental Table 12. β-coefficients and bootstrap inclusion frequencies of variables included in prediction models for all-cause dementia in the late-life cohort 20](#_Toc142463129)

[Supplemental Table 13. β-coefficients and bootstrap inclusion frequencies of variables included in prediction models for Alzheimer’s disease in the late-life cohort 21](#_Toc142463130)

[Supplemental Table 14. β-coefficients and bootstrap inclusion frequencies of variables included in prediction models for vascular dementia in the late-life cohort 22](#_Toc142463131)

[References 23](#_Toc142463132)


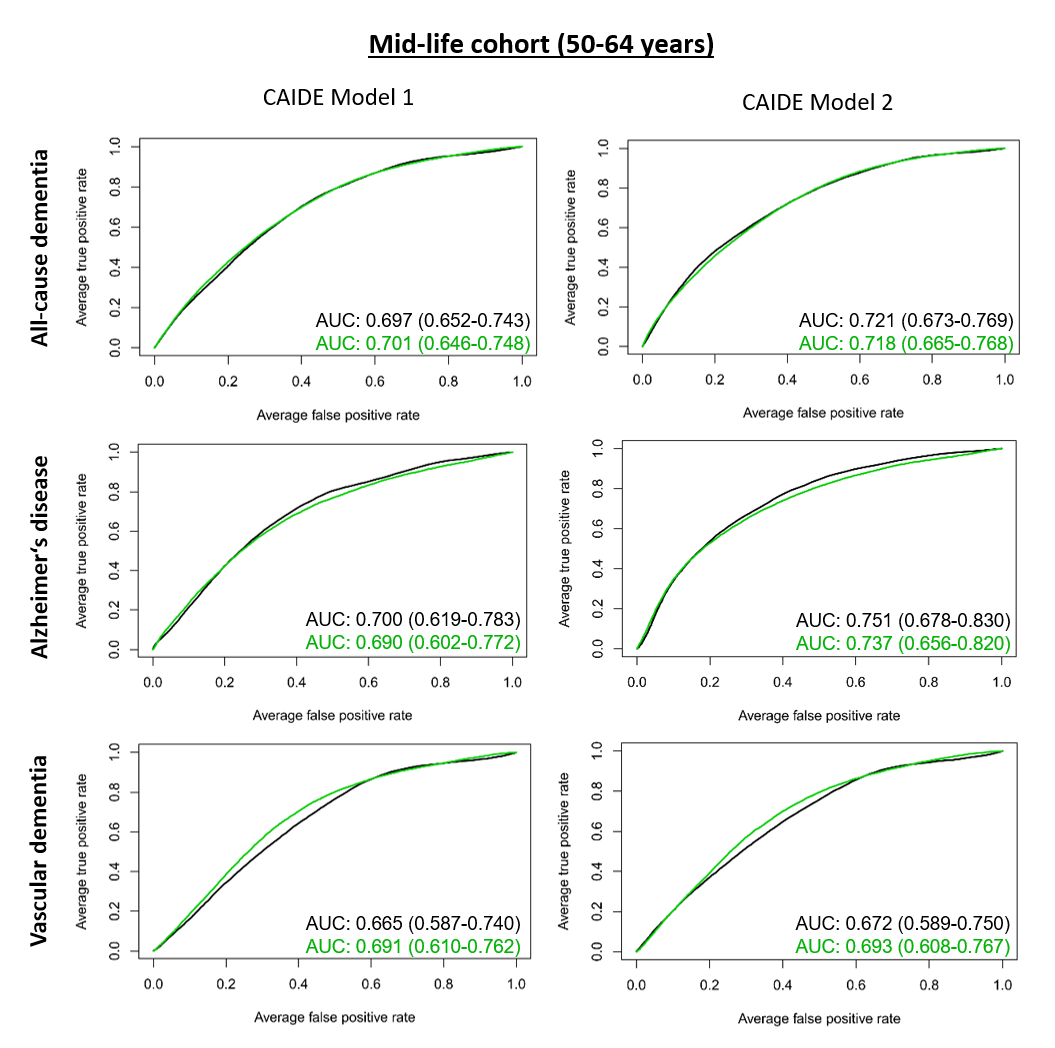
Supplemental Figure 1. ROC curves of created all-cause dementia, Alzheimer’s disease and vascular dementia risk prediction models for the mid-life cohort.

ROC curves for CAIDE model 1 (including age, education, sex, systolic blood pressure, BMI, total cholesterol, and physical activity, and CAIDE model 2 (additionally including *APOE* ε4 carrier status) are depicted in black while curves of the CAIDE models plus inflammatory biomarkers chosen by LASSO regression (cf. Supplemental Tables 9-11) are depicted in green. AUC and 95% confidence intervals are provided with the respective graphs. The AUCs were obtained in a nested case-cohort study with n=867 healthy controls and n=207, n=65, and n=68 cases for all-cause dementia, Alzheimer’s disease, and vascular dementia, respectively.

Abbreviations: BMI, body mass index, APOE, apolipoprotein; LASSO, least absolute shrinkage and selection operator


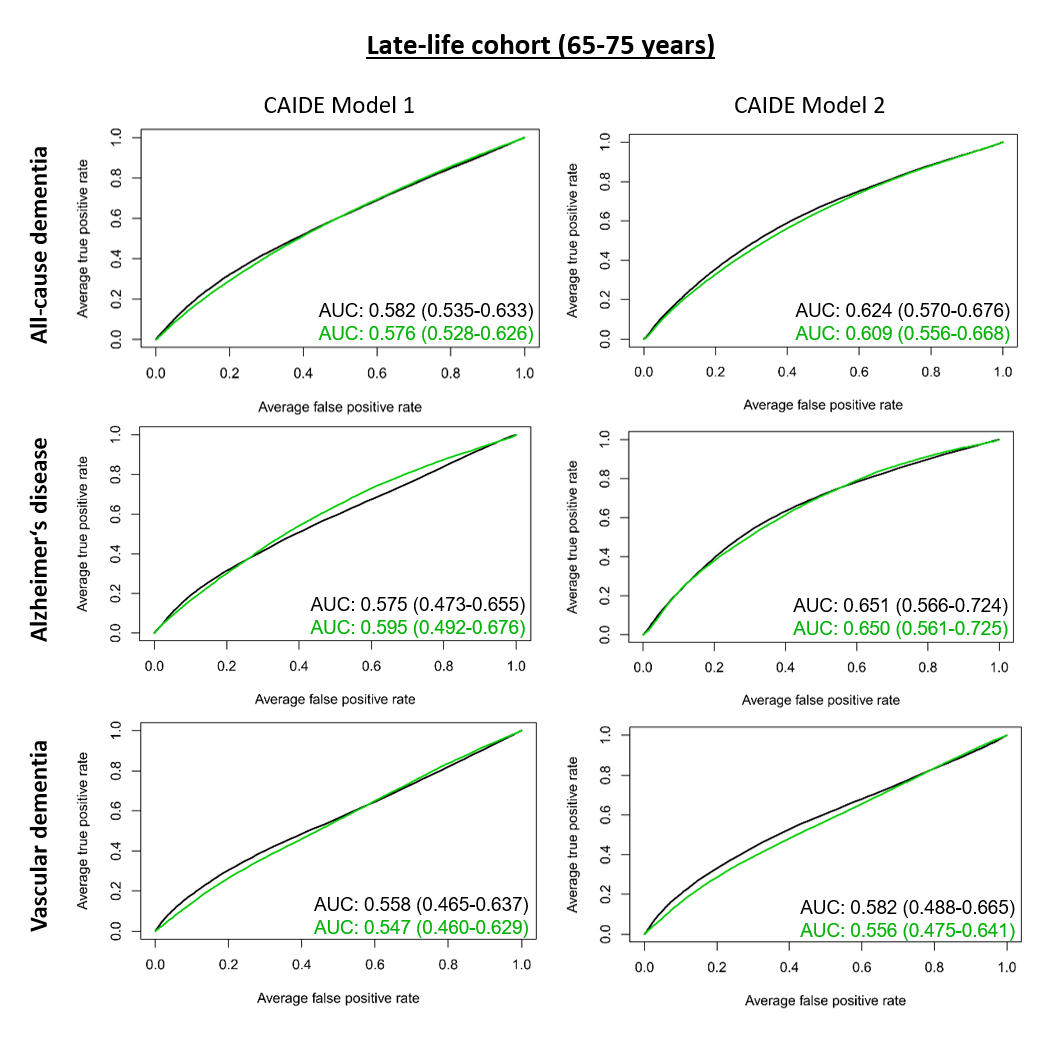
Supplemental Figure 2. ROC curves of created all-cause dementia, Alzheimer’s disease and vascular dementia risk prediction models for the late-life cohort.

ROC curves for CAIDE model 1 (including age, education, sex, systolic blood pressure, BMI, total cholesterol, and physical activity, and CAIDE model 2 (additionally including *APOE* ε4 carrier status) are depicted in black while curves of the CAIDE models plus inflammatory biomarkers chosen by LASSO regression (cf. Supplemental Tables 12-14) are depicted in green. AUC and 95% confidence intervals are provided with the respective graphs. The AUCs were obtained in a nested case-cohort study with n=489 healthy controls and n=355, n=108, and n=131 cases for all-cause dementia, Alzheimer’s disease, and vascular dementia, respectively.

Abbreviations: BMI, body mass index, APOE, apolipoprotein; LASSO, least absolute shrinkage and selection operator

# Supplemental Text 1. Quantative biomarker analysis

Inflammation-related, blood-based proteins were measured from serum samples collected during the health checkup at baseline (2000-2002). Blood samples were sent to the study centre and stored at -80°C until biomarker measurements took place in March 2018, December 2018, September 2020, and April 2023 (referred to as time points t1, t2, t3, and t4 in the following). At the time of the measurements, 10-25 µl of serum was extracted from different aliquots that had been thawed twice and sent on dry ice to the laboratories, which analyzed the samples with the Olink Target 96 Inflammation panel, Olink Proteomics, Uppsala, Sweden. At t1 and t2, samples were analyzed in the laboratory of Olink Proteomics, Uppsala Science Park, SE-75183 Uppsala, Sweden. At t3 and t4, the measurements were performed in the Metabolomics and Proteomics Core, German Research Center for Environmental Health, Helmholtz Center Munich, Heidemannstraße 1, 80939 München, Germany.

The Olink panels are based on a Proximity Extension Assay technology (PEA)[1, 2]. Details on the reliability and stability of the technology are described elsewhere[3]. In brief, oligonucleotide labelled antibody probe pairs are allowed to bind to their respective target proteins in the samples. Only if two antibodies are in close proximity, annealing of the oligonucleotides is enabled and a polymerase chain reaction (PCR) reporter sequence is formed by DNA polymerization. This sequence is detected and quantified using high throughput real-time quantitative PCR (qPCR) (Fluidigm® Biomark^TM^ HD system). The Olink Target 96 Inflammation panel allows the measurement of 92 biomarkers per sample. A list of all biomarkers of this panel is displayed in **Supplemental Table 2**.

At t1, t2, t3, and t4, 22, 15, 5, and 4 plates were used, respectively. To avoid batch effects, cases and controls were randomly distributed across plates and adjusted according to included interpolate controls. The average intra-assay coefficient of variance among all 92 measured biomarkers was 7%, 4%, 3%, and 5% at t1, t2, t3, and t4, respectively. The average inter-assay coefficient of variance was 12%, 10%, 10%, and 13% at t1, t2, t3, and t4, respectively. Furthermore, the quality of each serum sample was assessed by Olink technology[4]. All samples were measured successfully, and the number of quality control warnings was below 4% in the first three timepoints and below 10% in t4. Of the 1,496 randomly selected controls and 656 incident dementia cases, 75 serum samples of participants were excluded due to a quality control warning .

Protein levels are reported as Normalized Protein eXpression (NPX) values, a relative quantification unit logarithmically related to protein concentration. The number of samples with values below the lower limit of detection (LOD) varied strongly by biomarker and is shown in **Supplemental Table 2** (Appendix). In total, 23 biomarkers with > 25 % of the values below LOD were excluded from all analyses (grey shaded biomarkers in Supplemental Table 2). Thereby, 69 out of the 92 biomarkers were considered evaluable markers. Biomarker values below the LOD were replaced by LOD/√2. The normalization of raw data was conducted with the R (R Core Team, 2020, version 3.6.3) package “OlinkAnalyze”, developed and maintained by the Olink Proteomics Data Science Team [5]. For this procedure, bridging samples were used to normalize data from three different measurement time points.

Supplemental Table 1. Comparison of age, education, and sex of included and excluded study participants of the ESTHER study

| **CAIDE model variables** | **Excluded study participants (n=145)** | **Included study participants (n=1637)** |
| --- | --- | --- |
| **Age (years), mean (SD)** | 64.5 (6.7) | 63.1 (6.5) |
| **Education (years), n (%)** |  |  |
| < 9 | 78 (70.3) | 1484 (77.4) |
| ≥ 9 | 33 (29.7) | 434 (22.6) |
| **Sex, n (%)** |  |  |
| Female | 82 (51.6) | 1044 (54.4) |
| Male | 77 (48.4) | 874 (45.6) |

Note: values are either presented as Mean (±SD) or in categories (n (%)).

Supplemental Table 2. Biomarkers measured with Olink Proseek® Multiplex Inflammation I96x96 kits.

| **Abbreviation** | **Biomarker name** | **Values < LOD** |
| --- | --- | --- |
| 4E-BP1 | Eukaryotic translation initiation factor 4E-binding protein 1 | 0% |
| ADA | Adenosine Deaminase | 0% |
| ARTN | Artemin | 81% |
| AXIN1 | Axin-1 | 7% |
| Beta-NGF | Beta-nerve growth factor | 18% |
| CASP-8 | Caspase-8 | 1% |
| CCL11 | Eotaxin | 0% |
| CCL19 | C-C motif chemokine 19 | 0% |
| CCL20 | C-C motif chemokine 20 | 0% |
| CCL23 | C-C motif chemokine 23 | 0% |
| CCL25 | C-C motif chemokine 25 | 0% |
| CCL28 | C-C motif chemokine 28 | 3% |
| CCL3 | C-C motif chemokine 3 | 0% |
| CCL4 | C-C motif chemokine 4 | 0% |
| CD244 | Natural killer cell receptor 2B4 | 0% |
| CD40 | CD40L receptor | 0% |
| CD5 | T-cell surface glycoprotein CD5 | 0% |
| CD6 | T cell surface glycoprotein CD6 isoform | 0% |
| CD8A | T-cell surface glycoprotein CD8 alpha chain | 0% |
| CDCP1 | CUB domain-containing protein 1 | 0% |
| CSF-1 | Macrophage colony-stimulating factor 1 | 0% |
| CST5 | Cystatin D | 4% |
| CX3CL1 | Fractalkine | 0% |
| CXCL1 | C-X-C motif chemokine 1 | 0% |
| CXCL10 | C-X-C motif chemokine 10 | 0% |
| CXCL11 | C-X-C motif chemokine 11 | 0% |
| CXCL5 | C-X-C motif chemokine 5 | 0% |
| CXCL6 | C-X-C motif chemokine 6 | 0% |
| CXCL9 | C-X-C motif chemokine 9 | 0% |
| DNER | Delta and Notch-like epidermal growth factor-related receptor | 0% |
| EN-RAGE | Protein S100-A12 | 0% |
| FGF-19 | Fibroblast growth factor 19 | 0% |
| FGF-21 | Fibroblast growth factor 21 | 0% |
| FGF-23 | Fibroblast growth factor 23 | 36% |
| FGF-5 | Fibroblast growth factor 5 | 43% |
| Flt3L | Fms-related tyrosine kinase 3 ligand | 0% |
| GDNF | Glial cell line-derived neurotrophic factor | 37% |
| HGF | Hepatocyte growth factor | 0% |
| IFN_gamma | Interferon gamma | 74% |
| IL1_alpha | Interleukin-1 alpha | 84% |
| IL-10 | Interleukin-10 | 5% |
| IL-10RA | Interleukin-10 receptor subunit alpha | 21% |
| IL-10RB | Interleukin-10 receptor subunit beta | 0% |
| IL-12B | Interleukin-12 subunit beta | 0% |
| IL13 | Interleukin-13 | 88% |
| IL-15RA | Interleukin-15 receptor subunit alpha | 30% |
| IL-17A | Interleukin-17A | 31% |
| IL-17C | Interleukin-17C | 37% |
| IL-18 | Interleukin-18 | 0% |
| IL-18R1 | Interleukin-18 receptor 1 | 0% |
| IL2 | Interleukin-2 | 98% |
| IL20 | Interleukin-20 | 82% |
| IL-20RA | Interleukin-20 receptor subunit alpha | 61% |
| IL22-RA1 | Interleukin-22 receptor subunit alpha-1 | 88% |
| IL24 | Interleukin-24 | 91% |
| IL2RB | Interleukin-2 receptor subunit beta | 75% |
| IL33 | Interleukin-33 | 97% |
| IL4 | Interleukin-4 | 85% |
| IL-5 | Interleukin-5 | 67% |
| IL-6 | Interleukin-6 | 1% |
| IL-7 | Interleukin-7 | 0% |
| IL-8 | Interleukin-8 | 0% |
| LAP TGF-beta-1 | Latency-associated peptide transforming growth factor beta-1 | 0% |
| LIF | Leukemia inhibitory factor | 68% |
| LIFR | Leukemia inhibitory factor receptor | 0% |
| MCP-1 | Monocyte chemotactic protein 1 | 0% |
| MCP-2 | Monocyte chemotactic protein 2 | 0% |
| MCP-3 | Monocyte chemotactic protein 3 | 8% |
| MCP-4 | Monocyte chemotactic protein 4 | 0% |
| MMP-1 | Matrix metalloproteinase-1 | 0% |
| MMP-10 | Matrix metalloproteinase-10 | 0% |
| NRTN | Neurturin | 75% |
| NT-3 | Neurotrophin-3 | 23% |
| OPG | Osteoprotegerin | 0% |
| OSM | Oncostatin-M | 1% |
| PD-L1 | Programmed cell death 1 ligand 1 | 0% |
| SCF | Stem cell factor | 0% |
| SIRT2 | SIR2-like protein 2 | 11% |
| SLAMF1 | Signaling lymphocytic activation molecule | 13% |
| ST1A1 | Sulfotransferase 1A1 | 6% |
| STAMBP | STAM-binding protein | 0% |
| TGF-alpha | Transforming growth factor alpha | 0% |
| TNF | Tumor necrosis factor | 66% |
| TNFB | TNF-beta | 0% |
| TNFRSF9 | Tumor necrosis factor receptor superfamily member 9 | 0% |
| TNFSF14 | Tumor necrosis factor ligand superfamily member 14 | 0% |
| TRAIL | TNF-related apoptosis-inducing ligand | 0% |
| TRANCE | TNF-related activation-induced cytokine | 0% |
| TSLP | Thymic stromal lymphopoietin | 84% |
| TWEAK | Tumor necrosis factor (Ligand) superfamily, member 12 | 0% |
| uPA | Urokinase-type plasminogen activator | 0% |
| VEGF-A | Vascular endothelial growth factor-A | 0% |

Note: Grey shade indicates biomarkers with 25% or more of the values below the lower limit of detection (LOD)

Supplemental Table 3. Associations of CAIDE model variables with all-cause dementia.

| **CAIDE model variables** | **CAIDE model 1**^a^ | |  | **CAIDE model 2**^b^ | |
| --- | --- | --- | --- | --- | --- |
|  | **Multivariate Odds Ratio (95%CI)^a^** | **χ^2^ test**  **p-value** |  | **Multivariate Odds Ratio (95%CI)^b^** | **χ^2^ test**  **p-value** |
| **Age (years), per 1 year** | **1.13 (1.11-1.15)** | **<0.0001** |  | **1.13 (1.11-1.15)** | **<0.0001** |
| **Education (years)** |  |  |  |  |  |
| < 9 | 1.00 Ref. |  |  | 1.00 Ref. |  |
| ≥ 9 | 1.00 (0.77-1.30) | 0.9973 |  | 0.97 (0.74-1.26) | 0.8017 |
| **Sex** |  |  |  |  |  |
| Female | 1.00 Ref. |  |  | 1.00 Ref. |  |
| Male | 1.02 (0.82-1.27) | 0.8673 |  | 1.04 (0.83-1.29) | 0.7387 |
| **Systolic blood pressure (mm Hg), per 1 mmHg** | 1.00 (1.00-1.01) | 0.1636 |  | 1.00 (1.00-1.01) | 0.1792 |
| **Body-mass index (kg/m^2^), per 1 kg/m²** | 0.98 (0.95-1.00) | 0.0633 |  | 0.98 (0.95-1.00) | 0.0942 |
| **Total cholesterol (mmol/L), per 1 mmol/L** | **0.87 (0.80-0.95)** | **0.0017** |  | **0.86 (0.79-0.94)** | **0.0005** |
| **Physical activity^c^** |  |  |  |  |  |
| Inactive | 1.00 Ref. |  |  | 1.00 Ref. |  |
| Active | **0.74 (0.57-0.96)** | **0.0213** |  | **0.75 (0.58-0.98)** | **0.0329** |
| ***APOE* genotypes** |  |  |  |  |  |
| ε4 non-carrier | - | - |  | 1.00 Ref. |  |
| ε4 carrier | - | - |  | **2.20 (1.76-2.76)** | **<0.0001** |

Note: Numbers printed in bold are statistically significant.

Abbreviations: CI, Confidence Interval; *APOE*, apolipoprotein E.

^a^The CAIDE model 1 includes age, education, sex, systolic blood pressure, body-mass index, total cholesterol and physical activity.

^b^The CAIDE model 2 includes the variables of CAIDE model 1 and *APOE* ε4 status.

^c^“Inactive” was defined by <1 hour of vigorous or <1 hour light physical activity per week. All other amounts of physical activity were grouped into the category “Active.”

Supplemental Table 4. Associations of CAIDE model variables with Alzheimer’s disease.

| **CAIDE model variables** | **CAIDE model 1^a^** | |  | **CAIDE model 2^b^** | |
| --- | --- | --- | --- | --- | --- |
|  | **Multivariate Odds Ratio (95%CI)^a^** | **χ^2^ test**  **p-value** |  | **Multivariate Odds Ratio (95%CI)^b^** | **χ^2^ test**  **p-value** |
| **Age (years), per 1 year** | **1.13 (1.09-1.16)** | **<0.0001** |  | **1.13 (1.10-1.17)** | **<0.0001** |
| **Education (years)** |  |  |  |  |  |
| ≤ 9 | 1.00 Ref. |  |  | 1.00 Ref. |  |
| > 9 | 0.94 (0.61-1.44) | 0.7768 |  | 0.91 (0.59-1.41) | 0.6822 |
| **Sex** |  |  |  |  |  |
| Female | 1.00 Ref. |  |  | 1.00 Ref. |  |
| Male | 0.93 (0.66-1.31) | 0.6727 |  | 0.98 (0.69-1.39) | 0.9213 |
| **SBP (mmHg), per 1 mmHg** | 1.01 (1.00-1.01) | 0.2711 |  | 1.01 (1.00-1.01) | 0.2863 |
| **BMI (kg/m^2^), per 1 kg/m²** | **0.96 (0.92-1.00)** | **0.0332** |  | **0.96 (0.92-1.00)** | **0.0479** |
| **Total cholesterol (mmol/L), per 1 mmol/L** | 0.88 (0.77-1.01) | 0.0673 |  | **0.87 (0.75-1.00)** | **0.0480** |
| **Physical activity^c^** |  |  |  |  |  |
| Inactive | 1.00 Ref. |  |  | 1.00 Ref. |  |
| Active | **0.57 (0.39-0.84)** | **0.0039** |  | **0.58 (0.39-0.85)** | **0.0057** |
| ***APOE* genotypes** |  |  |  |  |  |
| ε4 non-carrier | - | - |  | 1.00 Ref. |  |
| ε4 carrier | - | - |  | **3.14 (2.23-4.41)** | **<0.0001** |

Note: Numbers printed in bold are statistically significant.

Abbreviations: CI, Confidence Interval; APOE, apolipoprotein E; SBP, systolic blood pressure; BMI, body mass index.

^a^The CAIDE model 1 includes age, education, sex, systolic blood pressure, body-mass index, total cholesterol and physical activity.

^b^The CAIDE model 2 includes the variables of CAIDE model 1 and *APOE* ε4 status.

^c^“Inactive” was defined by <1 hour of vigorous or <1 hour light physical activity per week. All other amounts of physical activity were grouped into the category “Active.”

Supplemental Table 5. Associations of CAIDE model variables with vascular dementia.

| **CAIDE model variables** | **CAIDE model 1^a^** | |  | **CAIDE model 2^b^** | |
| --- | --- | --- | --- | --- | --- |
|  | **Multivariate Odds Ratio (95%CI)^a^** | **χ^2^ test**  **p-value** |  | **Multivariate Odds Ratio (95%CI)^b^** | **χ^2^ test**  **p-value** |
| **Age (years), per 1 year** | **1.14 (1.10-1.17)** | **<0.0001** |  | **1.14 (1.10-1.17)** | **<0.0001** |
| **Education (years)** |  |  |  |  |  |
| ≤ 9 | 1.00 Ref. |  |  | 1.00 Ref. |  |
| > 9 | 0.99 (0.67-1.48) | 0.9631 |  | 0.97 (0.65-1.44) | 0.8719 |
| **Sex** |  |  |  |  |  |
| Female | 1.00 Ref. |  |  | 1.00 Ref. |  |
| Male | 0.97 (0.71-1.34) | 0.8678 |  | 1.00 (0.72-1.38) | 0.9805 |
| **SBP (mmHg), per 1 mmHg** | 1.00 (0.99-1.01) | 0.5818 |  | 1.00 (0.99-1.01) | 0.6033 |
| **BMI (kg/m^2^), per 1 kg/m²** | 0.98 (0.94-1.02) | 0.3407 |  | 0.98 (0.95-1.02) | 0.3872 |
| **Total cholesterol (mmol/L), per 1 mmol/L** | **0.87 (0.76-0.99)** | **0.0313** |  | **0.87 (0.76-0.99)** | **0.0282** |
| **Physical activity^c^** |  |  |  |  |  |
| Inactive | 1.00 Ref. |  |  | 1.00 Ref. |  |
| Active | 0.83 (0.57-1.20) | 0.3181 |  | 0.84 (0.58-1.24) | 0.3838 |
| ***APOE* genotypes** |  |  |  |  |  |
| ε4 non-carrier | - | - |  | 1.00 Ref. |  |
| ε4 carrier | - | - |  | **1.72 (1.24-2.40)** | **0.0013** |

Note: Numbers printed in bold are statistically significant.

Abbreviations: CI, Confidence Interval; APOE, apolipoprotein E; SBP, systolic blood pressure; BMI, body mass index.

^a^The CAIDE model 1 includes age, education, sex, systolic blood pressure, body-mass index, total cholesterol and physical activity.

^b^The CAIDE model 2 includes the variables of CAIDE model 1 and *APOE* ε4 status.

^c^“Inactive” was defined by <1 hour of vigorous or <1 hour light physical activity per week. All other amounts of physical activity were grouped into the category “Active.”

Supplemental Table 6. β-coefficients and bootstrap inclusion frequencies of variables included in prediction models for all-cause dementia in the total cohort

| **CAIDE model 1^a^** | | |  | **CAIDE model 2^b^** | | | |  |
| --- | --- | --- | --- | --- | --- | --- | --- | --- |
| **Variables** | **β-coefficient^c^** | **Bootstrap inclusion frequency (%)** |  | **Variables** | **β-coefficient^c^** | **Bootstrap inclusion frequency (%)** | |  |
| (Intercept) | -12.01159 | - |  | (Intercept) | -12.24676 | | - | |
| Age (per 1 year) | 0.12274 | 100.0 |  | Age (per 1 year) | 0.1271 | | 100.0 | |
| Education (high) | 0.04998 | 100.0 |  | Education (high) | 0.01896 | | 100.0 | |
| Sex (male) | 0.06769 | 100.0 |  | Sex (male) | 0.06596 | | 100.0 | |
| SBP (per 1 mmHg) | 0.00425 | 100.0 |  | SBP (per 1 mmHg) | 0.00402 | | 100.0 | |
| BMI (per 1 kg/m²) | -0.02642 | 100.0 |  | BMI (per 1 kg/m²) | -0.02605 | | 100.0 | |
| Total cholesterol (per 1 mmol/L) | -0.00324 | 100.0 |  | Total cholesterol (per 1 mmol/L) | -0.00369 | | 100.0 | |
| Physical activity (active) | -0.28056 | 100.0 |  | Physical activity (active) | -0.26459 | | 100.0 | |
| *APOE* genotypes (ε4 carrier) | - | - |  | *APOE* genotypes (ε4 carrier) | 0.81441 | | 100.0 | |
| Beta-NGF | -0.19681 | 44.2 |  | Beta-NGF | -0.24327 | | 52.8 | |
| CCL23 | -0.02279 | 11.4 |  | CCL23 | -0.07791 | | 13.0 | |
| - | - | - |  | CCL3 | -0.00897 | | 23.4 | |
| CD244 | 0.22513 | 80.6 |  | CD244 | 0.27415 | | 78.8 | |
| CXCL1 | -0.03556 | 20.2 |  | CXCL1 | -0.08025 | | 31.6 | |
| CXCL5 | 0.01078 | 18.4 |  | CXCL5 | 0.03535 | | 17.6 | |
| EN-RAGE | 0.12238 | 73.2 |  | EN-RAGE | 0.15417 | | 84.6 | |
| FGF-21 | 0.00554 | 27.0 |  | - | - | | - | |
| IL-18 | 0.04770 | 54.6 |  | IL-18 | 0.08782 | | 73.8 | |
| - | - | - |  | IL-7 | -0.02659 | | 14.4 | |
| LAP TGF-beta-1 | 0.12559 | 90.4 |  | LAP TGF-beta-1 | 0.14575 | | 88.2 | |
| LIFR | 0.03027 | 22.6 |  | LIFR | 0.12696 | | 36.4 | |
| - | - | - |  | MCP-3 | -0.00145 | | 26.0 | |
| OPG | 0.03039 | 39.8 |  | OPG | 0.00409 | | 28.0 | |
| OSM | -0.11366 | 36.0 |  | OSM | -0.10278 | | 31.6 | |
| - | - | - |  | SCF | -0.03946 | | 13.2 | |
| - | - | - |  | SIRT2 | -0.04784 | | 17.8 | |
| SLAMF1 | -0.07047 | 27.4 |  | SLAMF1 | -0.09037 | | 28.8 | |
| TNFB | -0.08117 | 30.0 |  | TNFB | -0.10107 | | 29.8 | |
| VEGF-A | 0.24644 | 99.8 |  | VEGF-A | 0.25251 | | 99.8 | |
| - | - | - |  | 4E-BP1 | 0.00761 | | 12.4 | |

Abbreviations: *APOE*, apolipoprotein E; SBP, systolic blood pressure; BMI, body mass index. For biomarker abbreviations, see Supplemental Table 2.

NOTE: Bootstrap inclusion frequencies for age, education, sex, SBP, BMI, total cholesterol, physical activity and APOE genotypes are always 100% because they were defined not to be penalized.

^a^The CAIDE model 1 includes age, education, sex, systolic blood pressure, body-mass index, total cholesterol and physical activity.

^b^The CAIDE model 2 includes the variables of CAIDE model 1 and *APOE* ε4 status.

^c^ß-coefficients shown for continuous variables are expressed per 1 unit. All variables except education, sex, physical activity and APOE genotype were modelled continuously. The categorical variables were dichotomized as shown in Supplemental Table 3.

Supplemental Table 7. β-coefficients and bootstrap inclusion frequencies of variables included in prediction models for Alzheimer’s disease in the total cohort

| **CAIDE model 1^a^** | | |  | **CAIDE model 2^b^** | | |
| --- | --- | --- | --- | --- | --- | --- |
| **Variables** | **β-coefficient^c^** | **Bootstrap inclusion frequency (%)** |  | **Variables** | **β-coefficient^c^** | **Bootstrap inclusion frequency (%)** |
| (Intercept) | -9.84785 | - |  | (Intercept) | -11.73183 | - |
| Age (per 1 year) | 0.12403 | 100 |  | Age (per 1 year) | 0.13413 | 100 |
| Education (high) | -0.04516 | 100 |  | Education (high) | 0.01062 | 100 |
| Sex (male) | 0.01426 | 100 |  | Sex (male) | 0.06600 | 100 |
| SBP (per 1 mmHg) | 0.00542 | 100 |  | SBP (per 1 mmHg) | 0.00467 | 100 |
| BMI (per 1 kg/m²) | -0.04183 | 100 |  | BMI (per 1 kg/m²) | -0.04424 | 100 |
| Total cholesterol (per 1 mmol/L) | -0.00343 | 100 |  | Total cholesterol (per 1 mmol/L) | -0.00457 | 100 |
| Physical activity (active) | -0.58639 | 100 |  | Physical activity (active) | -0.59848 | 100 |
| *APOE* genotypes (ε4 carrier) | - | - |  | *APOE* genotypes (ε4 carrier) | 1.25029 | 100 |
| CST5 | 0.02572 | 56.2 |  | CST5 | 0.01554 | 37.2 |
| EN-RAGE | 0.07672 | 90.8 |  | EN-RAGE | 0.16235 | 93.6 |
| - | - | - |  | IL-7 | -0.00815 | 23.8 |
| LAP TGF-beta-1 | 0.34050 | 99.2 |  | LAP TGF-beta-1 | 0.30830 | 97.0 |
| - | - | - |  | MCP-3 | -0.03683 | 53.0 |
| - | - | - |  | MMP-1 | 0.03126 | 44.2 |
| - | - | - |  | TRAIL | 0.07264 | 61.4 |

Abbreviations: *APOE*, apolipoprotein E; SBP, systolic blood pressure; BMI, body mass index. For biomarker abbreviations, see Supplemental Table 2.

NOTE: Bootstrap inclusion frequencies for age, education, sex, SBP, BMI, total cholesterol, physical activity and APOE genotypes are always 100% because they were defined not to be penalized.

^a^The CAIDE model 1 includes age, education, sex, systolic blood pressure, body-mass index, total cholesterol and physical activity.

^b^The CAIDE model 2 includes the variables of CAIDE model 1 and *APOE* ε4 status.

^c^ß-coefficients shown for continuous variables are expressed per 1 unit. All variables except education, sex, physical activity and APOE genotype were modelled continuously. The categorical variables were dichotomized as shown in Supplemental Table 3.

Supplemental Table 8. β-coefficients and bootstrap inclusion frequencies of variables included in prediction models for vascular dementia in the total cohort

| **CAIDE model 1^a^** | | |  | **CAIDE model 2^b^** | | |
| --- | --- | --- | --- | --- | --- | --- |
| **Variables** | **β-coefficient^c^** | **Bootstrap inclusion frequency (%)** |  | **Variables** | **β-coefficient^c^** | **Bootstrap inclusion frequency (%)** |
| (Intercept) | -10.64455 | - |  | (Intercept) | -10.49777 | - |
| Age (per 1 year) | 0.13493 | 100 |  | Age (per 1 year) | 0.13423 | 100 |
| Education (high) | 0.06336 | 100 |  | Education (high) | 0.03158 | 100 |
| Sex (male) | 0.00600 | 100 |  | Sex (male) | 0.04501 | 100 |
| SBP (per 1 mmHg) | 0.00121 | 100 |  | SBP (per 1 mmHg) | 0.00078 | 100 |
| BMI (per 1 kg/m²) | -0.01139 | 100 |  | BMI (per 1 kg/m²) | -0.00888 | 100 |
| Total cholesterol (per 1mmol/L) | -0.00260 | 100 |  | Total cholesterol (per 1mmol/L) | -0.00286 | 100 |
| Physical activity (active) | -0.15915 | 100 |  | Physical activity (active) | -0.12760 | 100 |
| *APOE* genotypes (ε4 carrier) | - | - |  | *APOE* genotypes (ε4 carrier) | 0.51857 | 100 |
| CD244 | 0.17163 | 84.8 |  | CD244 | 0.17842 | 85.8 |
| EN-RAGE | 0.08629 | 95.6 |  | EN-RAGE | 0.07665 | 94.0 |
| IL-18 | 0.08544 | 80.8 |  | IL-18 | 0.07970 | 84.4 |
| LAP TGF-beta-1 | 0.03588 | 57.4 |  | LAP TGF-beta-1 | 0.01516 | 56.0 |

Abbreviations: *APOE*, apolipoprotein E; SBP, systolic blood pressure; BMI, body mass index. For biomarker abbreviations, see Supplemental Table 2.

NOTE: Bootstrap inclusion frequencies for age, education, sex, SBP, BMI, total cholesterol, physical activity and APOE genotypes are always 100% because they were defined not to be penalized.

^a^The CAIDE model 1 includes age, education, sex, systolic blood pressure, body-mass index, total cholesterol and physical activity.

^b^The CAIDE model 2 includes the variables of CAIDE model 1 and *APOE* ε4 status.

^c^ß-coefficients shown for continuous variables are expressed per 1 unit. All variables except education, sex, physical activity and APOE genotype were modelled continuously. The categorical variables were dichotomized as shown in Supplemental Table 3.

Supplemental Table 9. β-coefficients and bootstrap inclusion frequencies of variables included in prediction models for all-cause dementia in the mid-life cohort

| **CAIDE model 1^a^** | | |  | **CAIDE model 2^b^** | | |
| --- | --- | --- | --- | --- | --- | --- |
| **Variables** | **β-coefficient^c^** | **Bootstrap inclusion frequency (%)** |  | **Variables** | **β-coefficient^c^** | **Bootstrap inclusion frequency (%)** |
| (Intercept) | -15.12894 | - |  | (Intercept) | -14.77237 | - |
| Age (per 1 year) | 0.19661 | 100 |  | Age (per 1 year) | 0.19638 | 100 |
| Education (high) | -0.03025 | 100 |  | Education (high) | -0.08746 | 100 |
| Sex (male) | 0.26896 | 100 |  | Sex (male) | 0.32143 | 100 |
| SBP (per 1 mmHg) | 0.00254 | 100 |  | SBP (per 1 mmHg) | 0.00125 | 100 |
| BMI (per 1 kg/m²) | -0.01800 | 100 |  | BMI (per 1 kg/m²) | -0.01764 | 100 |
| Total cholesterol (per 1 mmol/L) | -0.00450 | 100 |  | Total cholesterol (per 1 mmol/L) | -0.00533 | 100 |
| Physical activity (active) | -0.14135 | 100 |  | Physical activity (active) | -0.12558 | 100 |
| *APOE* genotypes (ε4 carrier) | - | - |  | *APOE* genotypes (ε4 carrier) | 0.86628 | 100 |
| CCL11 | 0.00398 | 32.2 |  | - | - | - |
| CXCL5 | 0.00309 | 44.6 |  | - | - | - |
| FGF-21 | 0.04874 | 79.0 |  | FGF-21 | 0.04105 | 74.2 |
| - | - | - |  | IL-18 | 0.01930 | 47.6 |
| - | - | - |  | IL-18R1 | 0.01016 | 44.0 |
| LIFR | 0.13884 | 57.8 |  | LIFR | 0.13904 | 61.8 |
| VEGF-A | 0.29162 | 99.8 |  | VEGF-A | 0.25433 | 99.0 |

Abbreviations: *APOE*, apolipoprotein E; SBP, systolic blood pressure; BMI, body mass index. For biomarker abbreviations, see Supplemental Table 2.

NOTE: Bootstrap inclusion frequencies for age, education, sex, SBP, BMI, total cholesterol, physical activity and APOE genotypes are always 100% because they were defined not to be penalized.

^a^The CAIDE model 1 includes age, education, sex, systolic blood pressure, body-mass index, total cholesterol and physical activity.
^b^The CAIDE model 2 includes the variables of CAIDE model 1 and *APOE* ε4 status.
^c^ß-coefficients shown for continuous variables are expressed per 1 unit. All variables except education, sex, physical activity and APOE genotype were modelled continuously. The categorical variables were dichotomized as shown in Supplemental Table 3.

Supplemental Table 10. β-coefficients and bootstrap inclusion frequencies of variables included in prediction models for Alzheimer’s disease in the mid-life cohort

| **CAIDE model 1^a^** | | |  | **CAIDE model 2^b^** | | |
| --- | --- | --- | --- | --- | --- | --- |
| **Variables** | **β-coefficient^c^** | **Bootstrap inclusion frequency (%)** |  | **Variables** | **β-coefficient^c^** | **Bootstrap inclusion frequency (%)** |
| (Intercept) | -15.76141 | - |  | (Intercept) | -19.01228 | - |
| Age (per 1 year) | 0.21075 | 100 |  | Age (per 1 year) | 0.21911 | 100 |
| Education (high) | -0.00800 | 100 |  | Education (high) | 0.02424 | 100 |
| Sex (male) | 0.24085 | 100 |  | Sex (male) | 0.37197 | 100 |
| SBP (per 1 mmHg) | 0.00846 | 100 |  | SBP (per 1 mmHg) | 0.00489 | 100 |
| BMI (per 1 kg/m²) | -0.02513 | 100 |  | BMI (per 1 kg/m²) | -0.03291 | 100 |
| Total cholesterol (per 1 mmol/L) | -0.00294 | 100 |  | Total cholesterol (per 1 mmol/L) | -0.00635 | 100 |
| Physical activity (active) | -0.74508 | 100 |  | Physical activity (active) | -0.60438 | 100 |
| *APOE* genotypes (ε4 carrier) | - | - |  | *APOE* genotypes (ε4 carrier) | 1.39512 | 100 |
| - | - | - |  | CCL20 | -0.07663 | 19.0 |
| CCL28 | 0.02115 | 40.8 |  | CCL28 | 0.04970 | 25.8 |
| - | - | - |  | CD6 | 0.07400 | 23.6 |
| - | - | - |  | CX3CL1 | 0.01586 | 15.0 |
| - | - | - |  | MCP-3 | -0.00633 | 15.4 |
| - | - | - |  | MMP-1 | 0.07550 | 21.8 |
| TRAIL | 0.45428 | 89.2 |  | TRAIL | 0.70757 | 88.0 |

Abbreviations: *APOE*, apolipoprotein E; SBP, systolic blood pressure; BMI, body mass index. For biomarker abbreviations, see Supplemental Table 2.

NOTE: Bootstrap inclusion frequencies for age, education, sex, SBP, BMI, total cholesterol, physical activity and APOE genotypes are always 100% because they were defined not to be penalized.

^a^The CAIDE model 1 includes age, education, sex, systolic blood pressure, body-mass index, total cholesterol and physical activity.

^b^The CAIDE model 2 includes the variables of CAIDE model 1 and *APOE* ε4 status.

^c^ß-coefficients shown for continuous variables are expressed per 1 unit. All variables except education, sex, physical activity and APOE genotype were modelled continuously. The categorical variables were dichotomized as shown in Supplemental Table 3.

Supplemental Table 11. β-coefficients and bootstrap inclusion frequencies of variables included in prediction models for vascular dementia in the mid-life cohort

| **CAIDE model 1^a^** | | |  | **CAIDE model 2^b^** | | |
| --- | --- | --- | --- | --- | --- | --- |
| **Variables** | **β-coefficient^c^** | **Bootstrap inclusion frequency (%)** |  | **Variables** | **β-coefficient^c^** | **Bootstrap inclusion frequency (%)** |
| (Intercept) | -22.04133 | - |  | (Intercept) | -21.90013 | - |
| Age (per 1 year) | 0.23425 | 100 |  | Age (per 1 year) | 0.23142 | 100 |
| Education (high) | 0.24376 | 100 |  | Education (high) | 0.21046 | 100 |
| Sex (male) | 0.18713 | 100 |  | Sex (male) | 0.28181 | 100 |
| SBP (per 1 mmHg) | -0.00800 | 100 |  | SBP (per 1 mmHg) | -0.00875 | 100 |
| BMI (per 1 kg/m²) | 0.01516 | 100 |  | BMI (per 1 kg/m²) | 0.01524 | 100 |
| Total cholesterol (per 1 mmol/L) | -0.00221 | 100 |  | Total cholesterol (per 1 mmol/L) | -0.00248 | 100 |
| Physical activity (active) | 0.12620 | 100 |  | Physical activity (active) | 0.09691 | 100 |
| *APOE* genotypes (ε4 carrier) | - | - |  | *APOE* genotypes (ε4 carrier) | 0.47880 | 100 |
| Beta-NGF | -0.49433 | 55.8 |  | Beta-NGF | -0.47838 | 57.0 |
| CD244 | 0.28237 | 74.4 |  | CD244 | 0.32216 | 79.2 |
| CXCL5 | 0.01746 | 33.2 |  | CXCL5 | 0.02229 | 36.2 |
| EN-RAGE | 0.10444 | 77.8 |  | EN-RAGE | 0.10727 | 78.8 |
| FGF-19 | 0.01716 | 37.0 |  | FGF-19 | 0.00673 | 33.8 |
| FGF-21 | 0.01525 | 32.0 |  | - | - | - |
| Flt3L | 0.33325 | 86.2 |  | Flt3L | 0.31739 | 84.2 |
| IL-18R1 | 0.14670 | 52.2 |  | IL-18R1 | 0.20361 | 63.0 |
| MMP-10 | 0.06590 | 48.2 |  | MMP-10 | 0.03765 | 37.6 |
| TNFRSF9 | 0.13497 | 61.4 |  | TNFRSF9 | 0.10437 | 58.0 |

Abbreviations: *APOE*, apolipoprotein E; SBP, systolic blood pressure; BMI, body mass index. For biomarker abbreviations, see Supplemental Table 2.

NOTE: Bootstrap inclusion frequencies for age, education, sex, SBP, BMI, total cholesterol, physical activity and APOE genotypes are always 100% because they were defined not to be penalized.

^a^The CAIDE model 1 includes age, education, sex, systolic blood pressure, body-mass index, total cholesterol and physical activity.

^b^The CAIDE model 2 includes the variables of CAIDE model 1 and *APOE* ε4 status.

^c^ß-coefficients shown for continuous variables are expressed per 1 unit. All variables except education, sex, physical activity and APOE genotype were modelled continuously. The categorical variables were dichotomized as shown in Supplemental Table 3.

Supplemental Table 12. β-coefficients and bootstrap inclusion frequencies of variables included in prediction models for all-cause dementia in the late-life cohort

| **CAIDE model 1^a^** | | |  | **CAIDE model 2^b^** | | |
| --- | --- | --- | --- | --- | --- | --- |
| **Variables** | **β-coefficient^c^** | **Bootstrap inclusion frequency (%)** |  | **Variables** | **β-coefficient^c^** | **Bootstrap inclusion frequency (%)** |
| (Intercept) | -6.02690 | - |  | (Intercept) | -5.79403 | - |
| Age (per 1 year) | 0.09559 | 100 |  | Age (per 1 year) | 0.10165 | 100 |
| Education (high) | 0.12805 | 100 |  | Education (high) | 0.08051 | 100 |
| Sex (male) | -0.12286 | 100 |  | Sex (male) | -0.12615 | 100 |
| SBP (per 1 mmHg) | 0.00429 | 100 |  | SBP (per 1 mmHg) | 0.00471 | 100 |
| BMI (per 1 kg/m²) | -0.04459 | 100 |  | BMI (per 1 kg/m²) | -0.04041 | 100 |
| Total cholesterol (per 1 mmol/L) | -0.00261 | 100 |  | Total cholesterol (per 1 mmol/L) | -0.00302 | 100 |
| Physical activity (active) | -0.40059 | 100 |  | Physical activity (active) | -0.38571 | 100 |
| *APOE* genotypes (ε4 carrier) |  | - |  | *APOE* genotypes (ε4 carrier) | 0.73568 | 100 |
| CD244 | 0.00491 | 40.0 |  | - | - | - |
| EN-RAGE | 0.04566 | 73.6 |  | - | - | - |
| LAP TGF-beta-1 | 0.07423 | 66.4 |  | - | - | - |

Abbreviations: *APOE*, apolipoprotein E; SBP, systolic blood pressure; BMI, body mass index. For biomarker abbreviations, see Supplemental Table 2.

NOTE: Selection frequencies for age, education, sex, SBP, BMI, total cholesterol, physical activity and APOE genotypes are always 100% because they were defined not to be penalized.

In the late-life sample, none of the inflammatory biomarkers was selected by LASSO regression for a potentially improved prediction of all-cause dementia with CAIDE model 2 and prediction of VD with CAIDE model 1.

^a^The CAIDE model 1 includes age, education, sex, systolic blood pressure, body-mass index, total cholesterol and physical activity.

^b^The CAIDE model 2 includes the variables of CAIDE model 1 and *APOE* ε4 status.

^c^ß-coefficients shown for continuous variables are expressed per 1 unit. All variables except education, sex, physical activity and APOE genotype were modelled continuously. The categorical variables were dichotomized as shown in Supplemental Table 3.

Supplemental Table 13. β-coefficients and bootstrap inclusion frequencies of variables included in prediction models for Alzheimer’s disease in the late-life cohort

| **CAIDE model 1^a^** | | |  | **CAIDE model 2^b^** | | |
| --- | --- | --- | --- | --- | --- | --- |
| **Variables** | **β-coefficient^c^** | **Bootstrap inclusion frequency (%)** |  | **Variables** | **β-coefficient^c^** | **Bootstrap inclusion frequency (%)** |
| (Intercept) | -6.91703 | - |  | (Intercept) | -8.46439 | - |
| Age (per 1 year) | 0.10402 | 100 |  | Age (per 1 year) | 0.12346 | 100 |
| Education (high) | -0.10781 | 100 |  | Education (high) | -0.10909 | 100 |
| Sex (male) | -0.31072 | 100 |  | Sex (male) | -0.27230 | 100 |
| SBP (per 1 mmHg) | 0.00304 | 100 |  | SBP (per 1 mmHg) | 0.00361 | 100 |
| BMI (per 1 kg/m²) | -0.06695 | 100 |  | BMI (per 1 kg/m²) | -0.06035 | 100 |
| Total cholesterol (per 1 mmol/L) | -0.00403 | 100 |  | Total cholesterol (per 1 mmol/L) | -0.00399 | 100 |
| Physical activity (active) | -0.46111 | 100 |  | Physical activity (active) | -0.52545 | 100 |
| *APOE* genotype (ε4 carrier) | - | - |  | *APOE* genotype (ε4 carrier) | 1.10369 | 100 |
| EN_RAGE | 0.17226 | 94.2 |  | EN-RAGE | 0.15592 | 94.2 |
| LAP TGF-beta-1 | 0.16066 | 79.4 |  | LAP TGF-beta-1 | 0.12304 | 76.8 |
| - | - | - |  | ST1A1 | 0.00457 | 42.2 |

Abbreviations: *APOE*, apolipoprotein E; SBP, systolic blood pressure; BMI, body mass index. For biomarker abbreviations, see Supplemental Table 2.

NOTE: Bootstrap inclusion frequencies for age, education, sex, SBP, BMI, total cholesterol, physical activity and APOE genotypes are always 100% because they were defined not to be penalized.

^a^The CAIDE model 1 includes age, education, sex, systolic blood pressure, body-mass index, total cholesterol and physical activity.

^b^The CAIDE model 2 includes the variables of CAIDE model 1 and *APOE* ε4 status.

^c^ß-coefficients shown for continuous variables are expressed per 1 unit. All variables except education, sex, physical activity and APOE genotype were modelled continuously. The categorical variables were dichotomized as shown in Supplemental Table 3.

Supplemental Table 14. β-coefficients and bootstrap inclusion frequencies of variables included in prediction models for vascular dementia in the late-life cohort

| **CAIDE model 1^a^** | | |  | **CAIDE model 2^b^** | | |
| --- | --- | --- | --- | --- | --- | --- |
| **Variables** | **β-coefficient^c^** | **Bootstrap inclusion frequency (%)** |  | **Variables** | **β-coefficient^c^** | **Bootstrap inclusion frequency (%)** |
| (Intercept) | -4.49544 | - |  | (Intercept) | -4.28267 | - |
| Age (per 1 year) | 0.08851 | 100 |  | Age (per 1 year) | 0.08180 | 100 |
| Education (high) | -0.00357 | 100 |  | Education (high) | -0.04949 | 100 |
| Sex (male) | -0.05355 | 100 |  | Sex (male) | -0.04132 | 100 |
| SBP (per 1 mmHg) | 0.00553 | 100 |  | SBP (per 1 mmHg) | 0.00563 | 100 |
| BMI (per 1 kg/m²) | -0.04767 | 100 |  | BMI (per 1 kg/m²) | -0.04555 | 100 |
| Total cholesterol (per 1 mmol/L) | -0.00355 | 100 |  | Total cholesterol (per 1 mmol/L) | -0.00367 | 100 |
| Physical activity (active) | -0.37768 | 100 |  | Physical activity (active) | -0.32339 | 100 |
| *APOE* genotype (ε4 carrier) | - | - |  | *APOE* genotypes (ε4 carrier) | 0.57808 | 100 |

Abbreviations: *APOE*, apolipoprotein E; SBP, systolic blood pressure; BMI, body mass index. For biomarker abbreviations, see Supplemental Table 2.

NOTE: Bootstrap inclusion frequencies for age, education, sex, SBP, BMI, total cholesterol, physical activity and APOE genotypes are always 100% because they were defined not to be penalized.

In the late-life sample, none of the inflammatory biomarkers was selected by LASSO regression for a potentially improved prediction of all-cause dementia with CAIDE model 2 and prediction of VD with CAIDE model 1.

^a^The CAIDE model 1 includes age, education, sex, systolic blood pressure, body-mass index, total cholesterol and physical activity.

^b^The CAIDE model 2 includes the variables of CAIDE model 1 and *APOE* ε4 status.

^c^ß-coefficients shown for continuous variables are expressed per 1 unit. All variables except education, sex, physical activity and APOE genotype were modelled continuously. The categorical variables were dichotomized as shown in Supplemental Table 3.

# References

1. Assarsson, E., et al., *Homogenous 96-plex PEA immunoassay exhibiting high sensitivity, specificity, and excellent scalability.* PLoS One, 2014. **9**(4): p. e95192.

2. Lundberg, M., et al., *Homogeneous antibody-based proximity extension assays provide sensitive and specific detection of low-abundant proteins in human blood.* Nucleic Acids Res, 2011. **39**(15): p. e102.

3. *Proseek® Multiplex Inflammation I^96x96^* [white paper] 2016 25 June 2020]; Available from: <https://www.olink.com/content/uploads/2015/12/0993-v1.1-Proseek-Multiplex-Inflammation-I-Data-Package_final.pdf>.

4. *Data normalization and standardization*. [white paper] 2018 25 June 2020]; Available from: <https://www.olink.com/content/uploads/2018/05/Data-normalization-and-standardization_v1.0.pdf>.

5. Team, O.P.D.S. *OlinkAnalyze*. 2018 07/03/2021]; Available from: <https://github.com/Olink-Proteomics/OlinkRPackage/tree/master/OlinkAnalyze>.
